# Sensing Cytosolic RpsL by Macrophages Induces Lysosomal Cell Death and Termination of Bacterial Infection

**DOI:** 10.1371/journal.ppat.1004704

**Published:** 2015-03-04

**Authors:** Wenhan Zhu, Lili Tao, Marsha L. Quick, Johanna A. Joyce, Jie-Ming Qu, Zhao-Qing Luo

**Affiliations:** 1 Department of Biological Sciences, Purdue University, West Lafayette, Indiana, United States of America; 2 Department of Pulmonary Medicine, Huadong Hospital, Shanghai Medical College, Fudan University, Shanghai, China; 3 Cancer Biology and Genetics Program, Memorial Sloan-Kettering Cancer Center, New York, New York, United States of America; Yale University School of Medicine, UNITED STATES

## Abstract

The intracellular bacterial pathogen *Legionella pneumophila* provokes strong host responses and has proven to be a valuable model for the discovery of novel immunosurveillance pathways. Our previous work revealed that an environmental isolate of *L*. *pneumophila* induces a noncanonical form of cell death, leading to restriction of bacterial replication in primary mouse macrophages. Here we show that such restriction also occurs in infections with wild type clinical isolates. Importantly, we found that a lysine to arginine mutation at residue 88 (K88R) in the ribosome protein RpsL that not only confers bacterial resistance to streptomycin, but more importantly, severely attenuated the induction of host cell death and enabled *L*. *pneumophila* to replicate in primary mouse macrophages. Although conferring similar resistance to streptomycin, a K43N mutation in RpsL does not allow productive intracellular bacterial replication. Further analysis indicated that RpsL is capable of effectively inducing macrophage death via a pathway involved in lysosomal membrane permeabilization; the K88R mutant elicits similar responses but is less potent. Moreover, cathepsin B, a lysosomal protease that causes cell death after being released into the cytosol upon the loss of membrane integrity, is required for efficient RpsL-induced macrophage death. Furthermore, despite the critical role of cathepsin B in delaying RpsL-induced cell death, macrophages lacking cathepsin B do not support productive intracellular replication of *L*. *pneumophila* harboring wild type RpsL. This suggests the involvement of other yet unidentified components in the restriction of bacterial replication. Our results identified RpsL as a regulator in the interactions between bacteria such as *L*. *pneumophila* and primary mouse macrophages by triggering unique cellular pathways that restrict intracellular bacterial replication.

## Author Summary

The death of the host cell during infection can be triggered by one or more microbial molecules; this “live or die” selection provides effective means for the dissection of immune recognition mechanisms as well as for the identification of the microbial molecules responsible for such responses. We found that infection of primary mouse macrophages by *Legionella pneumophila* strains harboring wild type RpsL, the S12 component of the bacterial ribosome, causes macrophage death by a mechanism independent of the three inflammatory caspases, caspase 1, 7 and 11. Importantly, although both confer resistance to streptomycin at indistinguishable effectiveness, the K88R, but not the K43N mutation in RpsL enables *L*. *pneumophila* to replicate in macrophages. Purified RpsL and RpsL_K43N_ physically delivered into macrophages cause cell death by inducing damage to lysosomal membranes and the release of cathepsins. We also found that the lysosomal protease cathepsin B is required for efficient RpsL-induced cell death but its absence is not sufficient for macrophages to support intracellular bacterial replication. Thus, RpsL functions as an immune induction molecule to trigger one or more signaling cascades that leads to lysosomal cell death as well as the termination of bacterial replication.

## Introduction

Pattern recognition receptors (PRRs) sense pathogen-associated molecular patterns (PAMPs) or damage-associated molecular patterns (DAMPs) generated by infection or endogenous cellular injury or tissue damage to initiate immune responses [1]. The Toll-like receptors (TLRs) were the first identified PRRs that recognize PAMPs and induce the expression of pro-death cytokines and pro-inflammatory molecules through the nuclear factor κB (NF-κB) signaling pathway [1]. These molecules could orchestrate efficient defense against invading pathogens through the induction of cell death, which is an effective means of defense against infections in many microbe-host interaction systems. For example, TNF-α engages the cellular apoptosis or necroptosis pathway to defend against infection [1]. The second group of PRRs contains the NOD-like receptor (NLR), the retinoic-acid inducible gene-I (RIG-I)-like helicase, and the PYHIN (pyrin and HIN200 domain—containing proteins; also known as p200 or HIN200 proteins) protein families [2]. These structurally and functionally heterologous proteins recognize more diverse ligands (including PAMPs) and can be generally divided into two categories based on their downstream signaling events. The first category of receptors promote transcriptional activation of cytokines through pathways controlled by the transcriptional activator NF-κB or IRF3 [2], whereas the second group of receptors initiate the assembly of large cytoplasmic signaling complexes known as the inflammasomes [2]. The inflammasome senses microbial infection and/or danger-associated molecules and activate caspase-1/11-dependent cytokine production and inflammatory cell death (pyroptosis), which is believed to be important in the removal of the replicative niche of intracellular pathogens [3].

Recent studies have identified the ligands for several of these receptors. For example, NAIP5 and NAIP6 are the receptors for bacterial flagellin, and NAIP1 and NAIP2 sense the needle and rod proteins of bacterial type III secretion systems, respectively [4–6]. Cytosolic DNA directly binds to the AIM2 inflammasome [7–9] and intracellular lipopolysaccharides (LPS) directly activate caspase 11, a key component of inflammatory response [10]. However, the ligands for other members of the NAIP family of NLR proteins remain elusive. Similarly, our understanding of the broader mechanisms underlying infection-induced death of immune cells is limited [11].

The pathogen *Legionella pneumophila* replicates within amoebae hosts in the environment; it also is able to grow in alveolar macrophages in the human lung, which causes Legionnaires’ disease. Intracellular replication of *L*. *pneumophila* requires the Dot/Icm type IV secretion system, which delivers hundreds of proteins into host cells to construct a niche supportive of bacterial growth [12–14]. Because its primary evolutionary pressure for virulence derives from life in amoebae hosts, *L*. *pneumophila* does not seem to have evolved sophisticated immune-evasive mechanisms. As a result, challenge of immune cells such as macrophages with this bacterium leads to strong immune responses often not seen with more finely-adapted pathogens [15]. Thus, *L*. *pneumophila* has emerged as a powerful tool in dissecting novel host immune recognition mechanisms [16]. For example, characterization of mutants capable of replicating in macrophages from incompatible mice led to the identification of flagellin as the bacterial factor responsible for triggering the strong immune response [17,18], which functioned by directly engaging NAIP5 to activate the NLRC4 inflammasome [4,5]. Similarly, mutants lacking effectors with similar biochemical activity have allowed the identification of protein synthesis inhibition as a signal for immune induction [19].

We recently found that an environmental *L*. *pneumophila* strain induces extensive cell death in mouse bone marrow-derived macrophages (BMDMs) that are permissive for commonly used laboratory strains [20]. Furthermore, the cell death differed from canonical apoptosis, necrosis or the caspase-1/11-dependent pyroptosis [20]. Here we found that clinical *L*. *pneumophila* strains induce similar responses. By identifying mutants capable of replicating in BMDMs, we found that the ribosomal protein RpsL is responsible for the restriction of replication. Although two mutations of RpsL both conferred bacterial resistance to the antibiotic streptomycin, one (K88R) allowed *L*. *pneumophila* isolates (both clinical and environmental) to grow in BMDMs, while the other (K43N) did not. Further cell biological studies revealed that RpsL induced cell death by triggering signaling cascades that led to lysosomal membrane permeabilization and subsequent cell death. Our results established RpsL as a ligand capable of triggering a unique immune recognition by inducing lysosomal cell death.

## Results

### A K88R mutation in *rpsL* allows strain LPE509 to replicate in mouse primary macrophages

**Fig 1:**
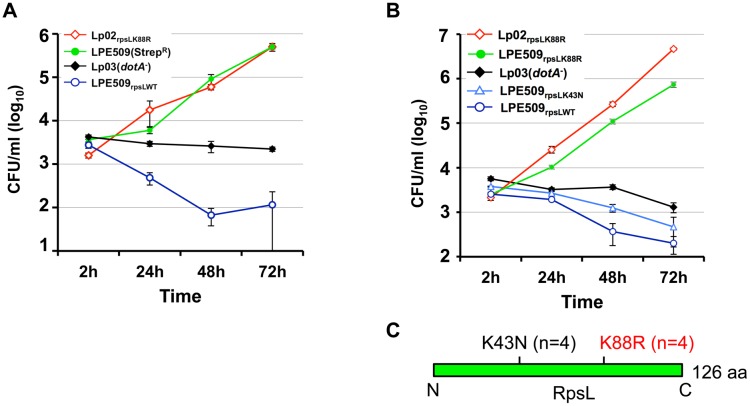
A K88R mutation in RpsL allows *L*. *pneumophila* strain LPE509 to replicate in macrophages. **A**. Intracellular growth of a spontaneous RpsL_K88R_ mutant of strain LPE509 in primary macrophages. **B**. K43N mutations do not allow *L*. *pneumophila* to replicate in macrophages. Bone marrow-derived macrophages from A/J mice were challenged with indicated bacterial strains at an MOI of 0.05. After synchronization 2 hrs postinfection, total bacterial counts were determined by plating appropriately diluted saponin solubilized infected cells onto bacteriological medium. Infections were performed in triplicate and data shown were from one representative of five experiments with similar results. **C**. Schematic diagram of the positions of the K43N and K88R mutations in RpsL, n is the number of independent mutants examined.

To approach the mechanism underlying cell death induction by strain LPE509 [20], we hypothesized that this strain codes for unique factor(s) capable of triggering a signaling cascade that leads to cell death, and that mutants lacking such factor(s) may enable the bacterium to replicate in macrophages. To identify such factor(s), we attempted to generate an insertion mutant library of LPE509 with a transposon introduced by bi-parental mating. To acquire an antibiotic resistance marker for eliminating the *Escherichia coli* donor cells used to deliver the transposon, we first isolated spontaneous streptomycin resistant (Strep^R^) mutants of LPE509, and the transposition mutant pools generated from two such mutants were then tested for intracellular replication in BMDMs from A/J mice, which are permissive for intracellular replication of laboratory *L*. *pneumophila* strains. Unexpectedly, robust intracellular growth was observed in all mutant pools derived from one particular Strep^R^ strain. Further analyses indicated that the parental strain used for mutant production had gained the ability to grow in BMDMs (Fig 1A). Sequencing analysis revealed a K88R mutation in the *rpsL* (lpg0324) gene of the Strep^R^ mutant. To rule out the possibility that additional mutations contributed to this phenotype, we introduced the *rpsL*_*K88R*_ allele into strain LPE509 by gene replacement to produce LPE509_rpsLK88R_; in BMDMs from A/J mice, this strain grew at rates comparable to the laboratory strain Lp02_rpsLK88R_, which increased almost 1000-fold in 72 hrs (Fig 1B). Consistent with earlier observations [20], wild type bacteria were cleared by macrophages within the experimental duration (Fig 1B). These results indicate that RpsL may regulate intracellular replication of *L*. *pneumophila* in BMDMs from A/J mice and that the gain-of-growth phenotype may result from the Strep^R^ property of the bacteria.

Because multiple mutations in the RpsL or the 16S rRNA can result in resistance to streptomycin [21], we assessed the intracellular growth of several independent streptomycin-resistant mutants for intracellular growth in BMDMs to determine whether all mutations that confer Strep^R^ result in the replication phenotype. Among 8 such mutants tested, 4 have overcome the restriction whereas the other 4 still were unable to replicate in macrophages (Figs 1B and S1). Sequencing analysis of the *rpsL* gene in these mutants revealed that in each of the 4 mutants capable of replicating in BMDMs, an A to G mutation occurred at nucleotide 263, causing a Lys to Arg substitution in the 88^th^ residue (K88R) of RpsL. In each of the 4 Strep^R^ mutants still defective in replicating in BMDMs, an A to T mutation at nucleotide 129 that led to a Lys to Asn substitution (K43N) in residue 43 was detected (Fig 1C). In agreement with the fact that the K88R and the K43N mutations confer indistinguishable resistance to streptomycin in bacteria such as *E*. *coli* [22], *L*. *pneumophila* mutants harboring these two mutations exhibited similar levels of streptomycin-resistance: both with a minimal inhibitory concentration (MIC) greater than 100 μg/ml (S1 Table). These results suggest that the K88R mutation in RpsL (RpsL_K88R_) is specific for overcoming the growth restriction of strain LPE509 in BMDMs permissive for commonly used laboratory *L*. *pneumophila* strains.

To further determine whether Strep^R^ *per se* is sufficient to permit intracellular growth in BMDMs, we introduced a plasmid expressing a streptomycin adenyltransferase [23] into strains LPE509. This strain has an MIC of 30 μg/ml (S1 Table), but cannot productively replicate in BMDMs (S2 Fig). Taken together, these results establish that a K88R mutation in the ribosome protein RpsL is sufficient to allow strain LPE509 to replicate in BMDMs and that resistance to Strep alone does not confer on *L*. *pneumophila* the ability to overcome the growth restriction imposed by these macrophages.

### RpsL is responsible for intracellular growth restriction of *L*. *pneumophila* in mouse primary macrophages

**Fig 2:**
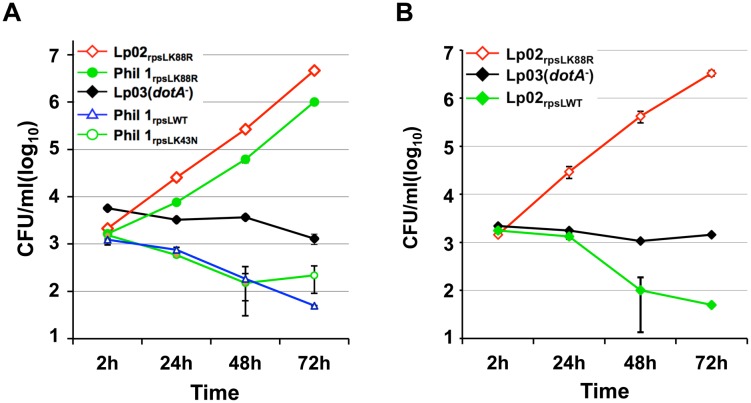
Wild type RpsL restricts the replication of *L*. *pneumophila* strains in macrophages. **A**. Strain Philadelphia 1 was unable to replicate in bone marrow-derived macrophages. **B**. Conversion of the *rpsL*_*K88R*_ allele of the laboratory strain Lp02_rpsLK88R_ into wild type abolished its ability to grow in macrophages. Experiments were performed in macrophages from A/J mice as described in Fig 1. With the exception of strains Lp02_rpsLK88R_ and Lp03, which harbor a spontaneous K88R mutation in *rpsL*, the *rpsL* locus of all other bacterial strains was introduced by genetic manipulation. Infections were performed in triplicate and data shown were from one representative of three experiments with similar results.

Our discovery that the RpsL_K88R_ mutation is required for *L*. *pneumophila* to grow in BMDMs is consistent with the fact that commonly used laboratory strains such as Lp02 [24] and JR32 [25] are resistant to streptomycin and that both harbor a K88R mutation in *rpsL* [26]. These results predicted that the parental streptomycin-sensitive strain Philadelphia 1 (Phil-1) [24] might be unable to replicate in BMDMs. Indeed, unlike its derivatives Lp02 (referred to as Lp02_rpsLK88R_ for clarity), strain Phil-1 was unable to replicate in BMDMs (Fig 2A). Importantly, robust growth occurred when the *rpsL* gene of strain Phil-1 was replaced by homologous recombination with *rpsL*_K88R_ but not with *rpsL*_K43N_ (Fig 2A). Similar growth restriction was observed in strains Paris [27] and Thunder Bay [28], in which a K88R mutation in RpsL allowed both strains to grow robustly in BMDMs (S3 Fig). Furthermore, strain Lp02_rpsLWT_ derived from Lp02_rpsLK88R_ has lost the ability to grow in BMDMs (Fig 2B). This strain, however, retained the ability to replicate in both Hela cells and the human macrophage cell line U937 (S4 Fig). Again, introduction of the streptomycin adenyltransferase gene into strain LPE509 or Lp02_rpsLWT_ conferred antibiotic resistance but not intracellular replication (S1 Table; S2 Fig), further supporting the notion that wild type RpsL is responsible for the restriction of bacterial replication in BMDMs. Taken together, these results indicate that wild type RpsL is the genetic determinant that governs the outcome of *L*. *pneumophila* infection in BMDMs; this protein restricts intracellular growth of *L*. *pneumophila* in BMDMs and that such restriction occurs in both clinical and environmental isolates. For clarity, we used strains Lp02_rpsLK88R_ and Lp02_rpsLWT_ in all subsequent experiments.

### Infection by *L*. *pneumophila* expressing wild type RpsL leads to macrophage death

**Fig 3:**
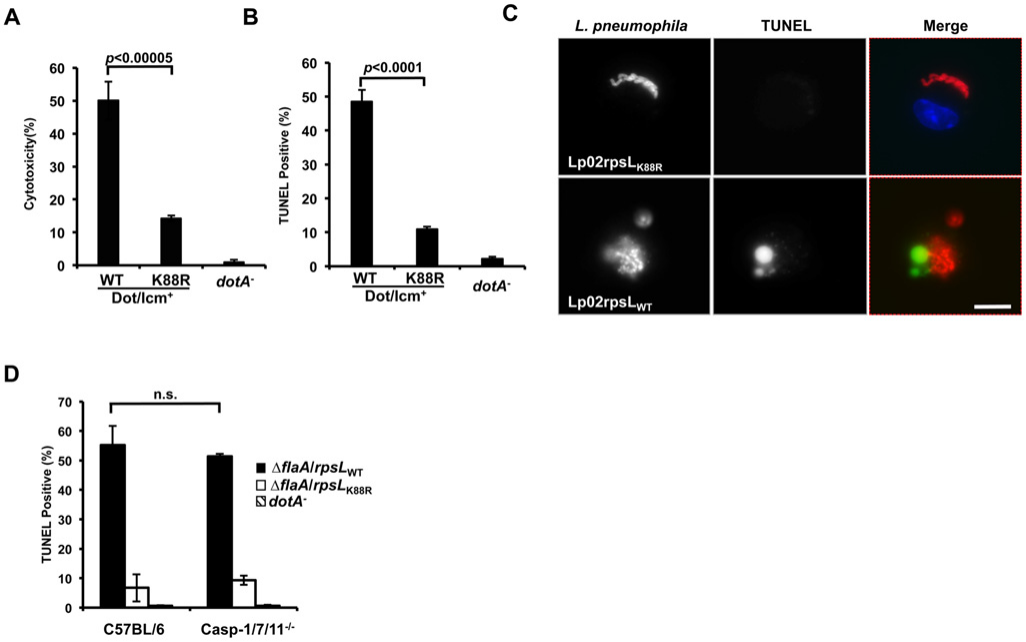
*L*. *pneumophila* strains harboring wild type *rpsL* caused cell death in infected macrophages. Bone marrow-derived macrophages from A/J mice were infected by the indicated bacterial strains for 14 hrs and health status of infected cells were evaluated by measuring the release of lactate dehydrogenase (LDH) (**A**) and by TUNEL staining (**B**) following by differently labeling extra- and intracellular bacterial by immunostaining. Infections were performed in triplicate and similar results were obtained in at least 3 independent experiments. For the TUNEL results, at least 300 infected cells were scored in each sample. **C**. Representative images of TUNEL signals and of bacterial phagosomes. Intracellular *L*. *pneumophila* bacteria were immunolabeled (red) with specific antibodies in BMDMs infected for 14 hrs; the status of host cell death was by TUNEL staining (green). Note the debris-like signals around the bacteria in samples infected with the strain harboring wild type RpsL. Images were acquired by a CCD camera with an Olympus IX-81 fluorescence microscope. Bar, 10 μm. **D**. Cell death caused by L. pneumophila occurs independent of the three inflammatory caspases, caspase-3, 7 and 11. BMDMs prepared from mice lacking caspases-3, 7 and 11 or its parental line C57BL/6 were infected with indicated *L*. *pneumophila* strains for 14 hrs and samples were processed to determine cell death as described in **B**. Note that the strain expressing wild type RpsL caused cell death in BMDMs from both mouse lines. The deletion of the *flaA* gene is to eliminate the effects caused by the NAIP5 allele in these mouse lines in response to flagellin.

Because host cell death accompanied the restriction of intracellular growth in infections with strain LPE509 [20], we determined whether strain Lp02_rpsLWT_ induces a similar phenotype. Compared to infections with strain Lp02_rpsLK88R_, significantly higher levels of lactate dehydrogenase (LDH) was detected in culture supernatant of BMDMs challenged with strain Lp02_rpsLWT_ (Fig 3A). Single cell analysis by the terminal deoxynucleotidyl transferase dUTP nick end labeling (TUNEL) staining revealed that at 12 hrs post-infection, close to 50% of the macrophages infected with Lp02_rpsLWT_ stained positively for apoptosis, whereas only about 10% Lp02_rpsLK88R_ infected cells appeared apoptotic (Fig 3B). At this time point, a fraction of the bacterial phagosomes have developed into vacuoles containing more than 10 bacteria. However, unlike Lp02_rpsLK88R_ that can be distinctly stained by the anti-*Legionella* antibody, the staining signals of Lp02_rpsLWT_ appeared diffuse, which could be the debris from dying bacterial cells detected by the polycolonal antibodies raised against fixed bacterial cells [29] (Fig 3C). Furthermore, although the presence of phagosomes with multiple bacteria is readily detectable in single cell analysis (Fig 3C and S5 Fig), the bacteria cells in these vacuoles appeared not to be viable as the total bacterial counts in samples infected with strains expressing wild type RpsL decreased at the 15 hrs or 24 hrs postinfection time points (Figs 1, 2 and S6).

Since inflammatory caspases such as caspase-1, 7 and 11 play important roles in macrophage death induced by bacteria infection [30,31], we thus examined whether these caspases play a role in the cell death induced by *L*. *pneumophila* expressing wild type RpsL. We infected macrophages from mice lacking caspase-1, 7 and 11 (caspase-1/7/11^-/-^) and found that the challenge of macrophages from these mice with strain Lp02_rpsLWT_∆*flaA* still led to cell death as measured by *TUNEL* staining. The reason to delete the *flaA* gene is that the genetic background of the knockout mice is C57BL/6, which is sensitive to flagellin produced in strain Lp02_rpsLWT_ [17,18]. Again, infection by strain Lp02rpsL_K88R_∆*flaA* caused significantly less death in these cells than did infection with strain Lp02_rpsLWT_∆*flaA* (Fig 3D). Consistent with the observation of cell death, BMDMs lacking caspase-1/7/11 did not support the growth of strain Lp02_rpsLWT_∆*flaA* (S7 Fig). Thus, infections with *L*. *pneumophila* expressing wild type RpsL cause macrophage death, which may contribute to the restriction of intracellular growth. Moreover, this cell death occurred in a mechanism independent of caspases-1, 7 and 11.

### RpsL directly induces cell death in macrophages

**Fig 4:**
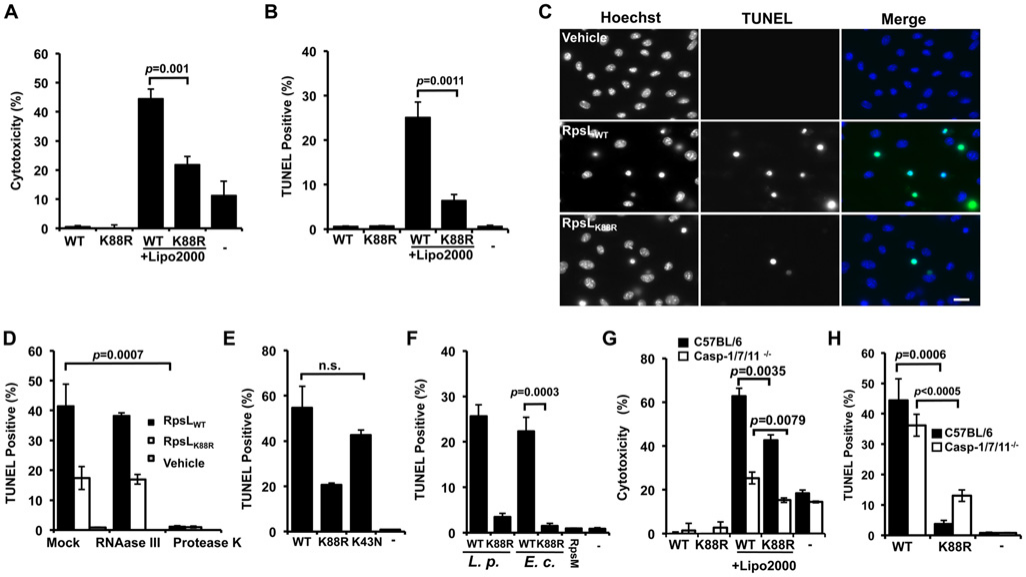
RpsL induces cell death in macrophage. **A–C**. Induction of cell death by RpsL. BMDMs from A/J mice were transfected with 10 μg purified RpsL or RpsL_K88R_ that had been washed with 60% isopropanol. 6 hrs posttransfection, samples were processed for measuring the level of LDH (**A**) or for TUNEL staining (**B**). Samples received the same amount of protein without the transfection reagent or treated with the transfection reagent only were set as controls. **C**. Representative images of the TUNEL staining of samples receiving RpsL or RpsL_K88R_. Note that adding the protein to the cell culture without the transfection reagent did not cause detectable cell death. Bar 10 μm. **D**. Protein but not dsRNA is responsible for the cell death. RNAase III or protease K was added to protein solution prior to transfection and samples were processed for TUNEL staining. **E–F**. Cell death induction by proteins relevant to RpsL. Macrophages transfected with RpsL, RpsL_K88R_, RpsL_K43N_, RpsM or RpsL from *E*. *coli* were processed for TUNEL staining. **G–H**. Macrophages from mice lacking caspases-1/7/11 are susceptible to cell death induced by RpsL. Macrophages prepared from the knockout or its parent mice were transfected with RpsL or RpsL_K88R_ and samples were processed for LDH release measurement (**G**) or for TUNEL staining (**H**). In all experiments, each treatment was performed in triplicate and the quantitative results were obtained from scoring at least 500 cells from each sample. Similar results were obtained from at least three independent experiments.-, transfection reagent only.

Because the K43N mutation in RpsL imposes higher translation accuracy on the ribosome [32,33], it is unlikely that the cell death is caused by factors such as the potential increase in mistranslated bacterial proteins. The high level of conservation of RpsL among taxonomically diverse bacteria prompted us to hypothesize that this protein is a ligand capable of inducing macrophage death. Thus, we purified recombinant His_6_-RpsL and His_6_-RpsL_K88R_ with a procedure employing the extensive isopropanol washing (methods) to exhaustively remove endotoxin (LPS) and delivered the proteins into macrophages by transfection [5]. When directly added to the BMDMs culture, neither protein caused detectable cell death (Fig 4A–B). However, when a transfection reagent was included, samples receiving His_6_-RpsL released significantly higher amount of LDH than samples receiving His_6_-RpsL_K88R_ (Fig 4A). Similar results were obtained when cell death was evaluated by TUNEL staining in which close to 30% of cells receiving wild type RpsL were apoptotic, which is significantly higher than the <10% seen in samples transfected with His_6_-RpsL_K88R_ (Fig 4B–C). Further, adding RNAase III to the protein did not alter its cell death induction activity, indicating that dsRNA [34] did not contribute to the observed phenotype (Fig 4D). On the other hand, treatment of protease K abolished the cell death-inducing ability of the protein (Fig 4D). Consistent with the fact that strains expressing RpsL_K43N_ cannot replicate within BMDMs, His_6_-RpsL_K43N_ induced macrophage death at rates comparable to those of wild type protein (Fig 4E). In addition, that wild type RpsL but not the K88R mutant from *E*. *coli* also induced robust cell death in macrophages (Fig 4F), which is consistent with the high-level conservation (91% identity) of this protein between these two bacteria. Furthermore, the cell death induction is specific for RpsL, as another similarly purified ribosomal protein, His_6_-RpsM was unable to induce macrophage death (Fig 4F). Finally, consistent with the observation that macrophage death induced by *L*. *pneumophila* strains harboring wild type RpsL is independent of caspases 1/7/11 (Fig 3D), His_6_-RpsL_WT_ induced cell death in macrophages lacking these caspases at rates comparable to those of C57BL/6 (Fig 4G–H). Because caspase-11 directly senses intracellular LPS [10], these results also indicate that the cell death was not caused by LPS potentially present in the RpsL protein preparations. Moreover, the fact that transfection is required for RpsL to exert its effects, this protein must be active only when it is present in the cytosol; this observation also suggested that RpsL was delivered into the cytoplasm of the host cell by *L*. *pneumophila* during infection. However, we were unable to detect such translocation using the β-lactamase reporter [12,35] (S8 Fig).

The fact that *L*. *pneumophila* strains harboring wild type *rpsL* were able to replicate in alternative hosts such as Hela and U937 cells (S4 Fig) suggested that RpsL did not induce death in these cells. Indeed, Hela cells transfected with His_6_-RpsL did not detectably stain positively for apoptosis (S9A Fig). The lack of cell death is not due to deficiency in protein delivery because a similar procedure successfully introduced purified β-galactosidase into these cells (S9B–S9C Fig). Taken together, these results establish that wild type RpsL directly induces cell death and such induction is specific for primary mouse macrophages; the K88R mutant triggers similar responses but in a less potent manner.

### RpsL causes lysosome membrane permeabilization in macrophages

**Fig 5:**
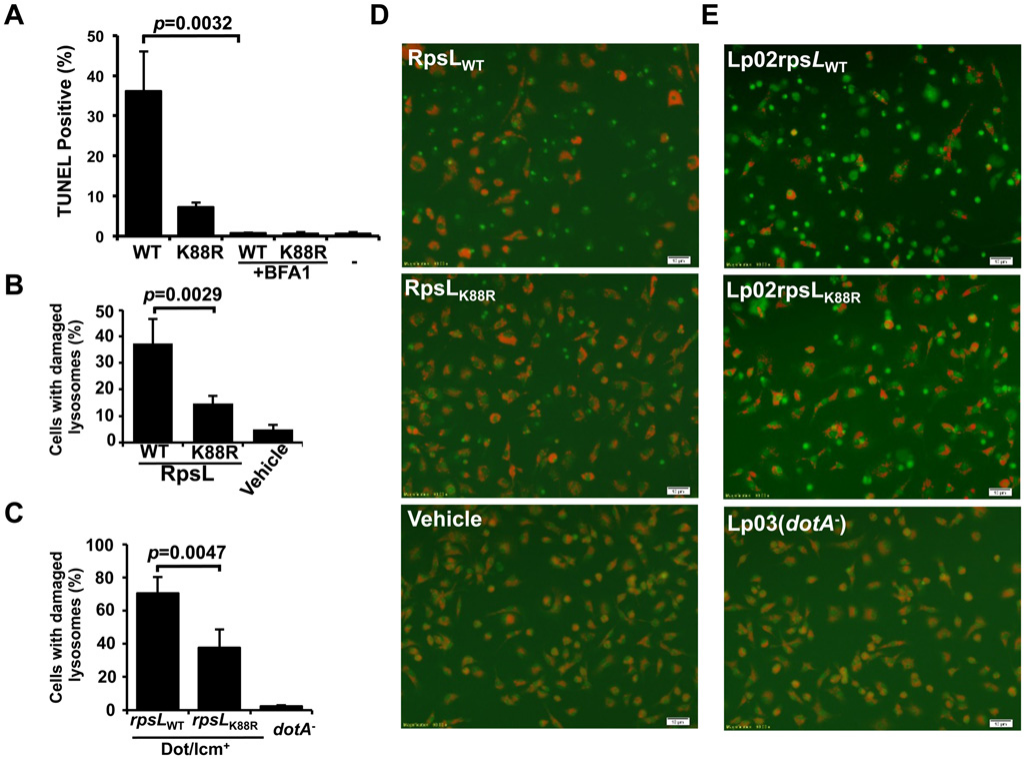
RpsL caused lysosome membrane permeabilization. **A**. Vacuole acidification is important for RpsL-induced macrophage death. Recombinant RpsL was transfected into BMDMs from A/J mice, bafilomycin A1 (20 nM), the inhibitor for vacuolar ATPase was added to a subset of samples. Cells fixed at 6 hrs after transfection were subjected for TUNEL staining. TUNEL signals were inspected and scored under a fluorescence microscope; at least 500 cells were examined in each sample performed in triplicate. **B**. RpsL caused lysosomal membrane damage. Purified RpsL or RpsL_K88R_ was delivered into BMDMs; after incubation in 5% CO_2_ at 37°C for 8 hrs, samples were centrifuged at 200*g* for 5 min, the culture medium was replaced with 0.5 ml of AO solution (5 μg/ml in PBS). The plate was incubated in 37°C for 15 min before imaging with a fluorescence microscope. Data shown are the percentage of cells with damaged lysosome (loss of red florescence) from one representative from three experiments with similar results. Quantitative results were obtained from at least 500 cells. **C**. Infection by *L*. *pneumophila* strain harboring wild type RpsL caused lysosomal membrane damage. BMDMs from A/J mice were challenged with indicated strains at an MOI of 1. Extracellular bacteria were removed with extensive wash 1 hr post infection, and cells were then stained with AO 7 hrs postinfection. The percentage of cells with ruptured lysosomes was quantitated from at 500 cells. All samples were performed in triplicate and similar results were obtained in at least 3 independent experiments. **D-E**. Representative images of AO fluorescence emission of BMDMs transfected with RpsL (**D**) or infected with relevant L. pneumophila strains (**E**).-, transfection reagent only. Bar, 10 μm.

Since macrophages from mice lacking the three inflammatory caspases are still sensitive to RpsL, it is very unlikely that the cell death is caused by canonical pyroptosis. We thus explored the involvement of other cell death pathways [36]. The lysosome, an organelle of low pH which contains a variety of hydrolases, has been implicated to play important roles in cell death in response to certain stimuli [37]. Such stimuli could trigger lysosome membrane permeabilization (LMP), leading to the release into the cytosol of hydrolases such as cathepsins (e.g. cathepsin B, D, and L) from the lysosomal lumen, where they induce apoptotic or pyroptotic cell death through caspase activation, or necroptosis when caspase activation was inhibited [38]. If RpsL activates the lysosomal cell death pathway, then inhibiting lysosomal acidification should reduce its cytotoxicity. To test this hypothesis, we inhibited lysosomal acidification by bafilomycin A1 (BFA1), which targets the vacuolar ATPase responsible for regulating organelle pH [39] prior to RpsL transfection. Cell death was almost completely abrogated in samples pre-incubated with BFA1 (Fig 5A).

We next assessed the integrity of lysosomal membranes in macrophages receiving RpsL using acridine orange (AO), a lysotrophic dye that when concentrated in lysosome, emits orange florescence upon blue light excitation, but green florescence when the loss of lysosome membrane integrity redistributes it into the cytosol and nucleus [40]. In samples transfected with RpsL, about 40% of the cells had lost lysosomal membrane integrity, as indicated by the emission of green fluorescence (Fig 5B&D); this dropped to approximately 15% of the cells when RpsL_K88R_ was delivered into the cells (Fig 5B&D). In controls not transfected with the protein, essentially all macrophages emitted orange but not green fluorescence (Fig 5B&D). Similar results were obtained in infections in which strain Lp02_rpsLWT_ caused significantly higher levels of lysosome damage than strain Lp02_rpsLK88R_ (Fig 5C&E). Taken together, we conclude that RpsL induces LMP.

### Cathepsin B is required for efficient RpsL-induced macrophage cell death

**Fig 6:**
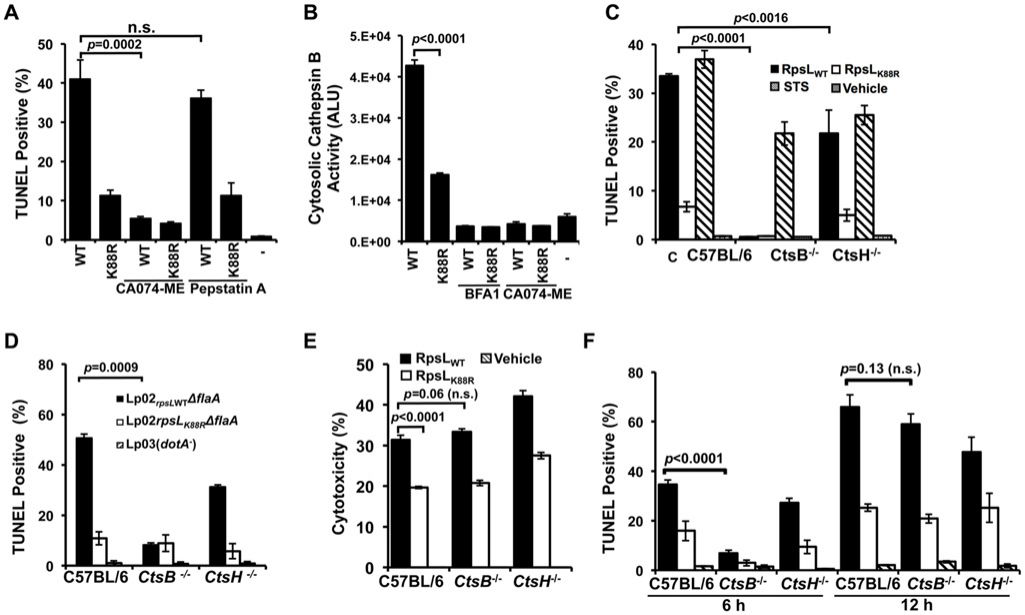
Cathepsin B is required for efficient RpsL-induced cell death in macrophages. **A**. Inhibition of CtsB activity alleviated RpsL-induced macrophage death. BMDMs from A/J mice were treated with 25-μM CA-074ME (against CtsB), 25-μM pepstatin A (against CtsD) or the solvent DMSO for 1.5 hrs before protein transfection. Cells were then processed for TUNEL staining. **B**. RpsL induced cytosolic release of cathepsin B. 1x10^5^ BMDMs in 96-well plates were transfected with the proteins for 8 hrs, and the cytosolic cathepsin activity was determined with the substrate zRR-AMC by measuring free AMC with a TECAN plate reader. **C**. Cathepsin B is required for RpsL-induced cell death at 6 hrs post protein transfection. BMDMs prepared from *ctsB* ^-/-^, *ctsH* ^-/-^ or C57B/6 mice were transfected with RpsL or RpsL_K88R_ for 6 hrs, and the cell death was evaluated by TUNEL staining. Samples treated with staurosporine were established as positive controls. **D**. Cell death induced by *L*. *pneumophila* expressing wild type RpsL required CtsB. BMDMs from the indicated mice were infected with relevant bacterial strains for 12 hrs at an MOI of 5 and cell death status of infected cells was determined by TUNEL staining. In both C and D, each treatment was performed in triplicate and TUNEL signals were assessed from at least 500 cells from each sample. Similar results were obtained in three independent experiments. **E**. RpsL caused plasma membrane damage in BMDMs from *ctsB* ^*-/-*^ mice. Macrophages from indicated mouse lines were transfected with RpsL or its K88R mutant for 6 hrs and the LDH release were then determined. Note the similar LDH release caused by RpsL among BMDMs from these mouse lines. **F**. BMDMs prepared from *ctsB* ^-/-^, *ctsH* ^-/-^ or C57B/6 mice transfected with RpsL or RpsL_K88R_ were fixed at 6 and 12 hrs post transfection, and the cell death was evaluated by TUNEL staining.-, transfection reagent only.

A direct result of LMP is the release of catabolic hydrolases, particularly cathepsin proteases, which have distinct roles in initiating cell death in different cell types [38]. We first used specific inhibitors to identify which cathepsin(s) are important for RpsL-induced cell death. CA074-Me, an inhibitor for cathepsin B (CtsB) but not pepstatin A, an inhibitor against cathepsin D antagonized RpsL-induced cell death (Fig 6A). Consistently, the cytosolic activity of cathepsins significantly increased upon RpsL delivery in a mechanism that required vacuolar acidification (Fig 6B). Notably, such increase also occurred in response to RpsL_K88R_ but at a significantly lower magnitude (Fig 6B). The strong inhibitory effects of CA074-Me on the cathepsin activity induced by RpsL suggest the selectivity of CtsB release, at least within our experimental duration.

To further examine the role of this protease in RpsL-induced cell death, we prepared macrophages from mice deficient in CtsB (*ctsb* ^-/-^) or CtsH (*ctsH* ^-/-^) and transfected them with recombinant RpsL. Six hours after transfection, cell death occurred in macrophages from both wild type and the *ctsH* ^-/-^ mice, although the rates in cells from the latter mouse line were significantly lower (Fig 6C). At the 6-hr time point, consistent with the abolishment of RpsL-induced cell death by inhibition of CtsB, almost no TUNEL positive cells were detected in macrophages from *ctsB* ^-/-^ mice (Fig 6C). These macrophages were not generally defective in apoptosis as staurosporine treatment induced extensive cell death (Fig 6C). Similar results were obtained in infections in which strain Lp02_rpsLWT_ induced significantly less cell death in BMDMs from *ctsB* ^-/-^ mice 14 hrs after uptake (Fig 6D). To determine whether BMDMs that received RpsL maintained their plasma membrane integrity, we measured the level of LDH in similarly treated samples. Intriguingly, although wild type RpsL is consistently more potent than the K88R mutant in inducing LDH release, similar levels of LDH were detected in BMDMs from the three different mouse lines after RpsL transfection (Fig 6E). In light of this observation, we determined the kinetics of RpsL-induced cell death in *ctsB* ^-/-^ BMDMs by TUNEL staining. Again, few TUNEL positive cells were detected in these cells within 6 hrs after transfection (Fig 6F). However, extensive cell death was observed in *ctsB* ^-/-^ BMDMs 12 hrs after transfection; at this time point, the rates of TUNEL positive cells among BMDMs from these three mouse lines became indistinguishable (Fig 6F). Consistent with these results, RpsL is able to induce the release of cathepsins such as aspartic cathepsin into the cytosol in *ctsB* ^-/-^ BMDMs (S10 Fig). These cathepsins may be responsible for the cell death observed when extended induction time was allowed after transfection. Taken together, these results establish that the lack of CtsB does not block but does significantly delays the cell death induced by RpsL.

**Fig 7:**
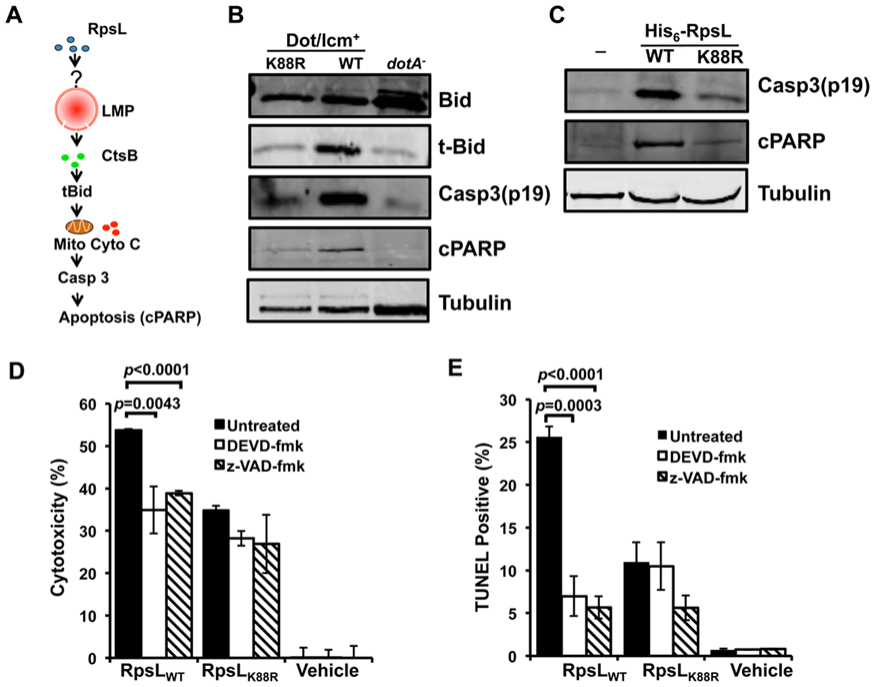
The classic apoptosis pathway is involved in the cell death induced by RpsL. **A**. A model for one of the possible cascades activated by RpsL that leads to macrophage death. Refer to the main text for details. **B–C**. The processing of apoptotic proteins by RpsL. BMDMs were infected with relevant bacterial strains with an MOI of 1 (**B**) or transfected with purified proteins (**C**) for 8 and 6 hrs, respectively. Samples were processed for immunoblot with specific antibodies. Tubulin was probed as a loading control. Similar results were obtained in at least two independent experiments. **D-E**. RpsL-induced macrophage death involves caspase activation. BMDMs from A/J mice were treated with 20-μM DEVD-fmk (against caspase-3), 20-μM z-VAD (against multiple caspases) or the solvent DMSO for 90 minutes before protein transfection. Six hours post protein transfection, LDH release (**D**) was determined or the samples were processed for TUNEL staining (**E**). Vehicle, transfection reagent only.

Cathepsins in the cytoplasm are able to activate cell death by cleaving the BH3-Only protein Bid to produce truncated Bid (tBid), which subsequently inserts into the mitochondrial membranes, leading to cytochrome c (Cyto c) release and caspase activation [41] (Fig 7A). Consistent with this notion, tBid was significantly more abundant in macrophages infected with strain Lp02_rpsLWT_ than in those infected with strain Lp02_rpsLK88R_ or samples infected with the *dotA* mutant (Fig 7B). Activation of caspase-3 and the subsequent cleavage of poly(ADP-ribose) polymerase (cPARP) also occurred more robustly in macrophages infected with Lp02_rpsLWT_ (Fig 7B). Caspase-3 is activated in permissive macrophages infected with strains harboring RpsL_K88R_ [12,42]; In agreement with the notion that RpsL induces cell death, such activation was significantly more robust in BMDMs infected with strains expressing wild type RpsL (Fig 7B). Furthermore, significantly more activated caspase-3 and cPARP can be detected in macrophages that received recombinant RpsL than those that received RpsL_K88R_ (Fig 7C). Consistent with the involvement of the activation of caspase-3 here, both DEVD-FMK, a cell-permeable, irreversible caspase-3-specific inhibitor [43] and the pan caspase inhibitor z-VAD-FMK [44] significantly dampened the RpsL-induced cell death within the first 6 hrs of protein transfection (Fig 7D–E). Thus, the canonical apoptosis pathway contributes to the cell death induced by RpsL.

We next examined the ability of BMDMs from *ctsB* ^-/-^ mice to support replication of the strain Lp02_rpsLWT_∆*fla*A. Consistent with earlier observations [17,18], deletion of *flaA* allowed strain Lp02_rpsLK88R_ to replicate in BMDMs from the C57BL/6 moue background. Despite the lack of cell death induced by strain Lp02_rpsLWT_∆*flaA* at 12 hrs post infection (Fig 6D), *ctsB* ^-/-^ BMDMs were unable to support intracellular growth of this strain (S11 Fig). This suggests that the cell death occurred after bacterial replication has been aborted or that the cell death is one of the branches of the signaling cascade induced by RpsL, which is consistent with the plasma membrane damage observed in *ctsB* ^-/-^ BMDMs that received recombinant RpsL (Fig 6E).

## Discussion

Genetically tractable pathogens are effective tools for probing host immune recognition mechanisms. By exploiting the cell death phenotype that restricts the replication of *L*. *pneumophila* in primary macrophages, we revealed that such cell death occurs in infections with essentially all wild type (streptomycin-sensitive) *L*. *pneumophila* strains, clinical or environmental. More importantly, we found that the ribosomal protein RpsL is directly responsible for such restriction. Our results supports a model in which RpsL activates a pathway that leads to lysosomal membrane permeabilization (LMP) and the subsequent apoptotic cell death, which is accompanied by the termination of intracellular bacteria replication.

It seems clear that RpsL is responsible for the macrophage death caused by *L*. *pneumophila* infection. A K88R mutation in RpsL allows the bacteria to replicate proficiently in these cells and the replacement of such mutations with the wild type allele in a widely used replication-competent laboratory strain abolished its ability to grow intracellularly in BMDMs. Although RpsL_K88R_ confers resistance to streptomycin, several lines of evidence indicate that this antibiotic apparently plays no role in intracellular replication. First, although the *rpsL*_K43N_ mutation confers resistance to streptomycin indistinguishably to that of the K88R mutation, mutants harboring RpsL_K43N_ were unable to grow in BMDMs. Second, expression of a streptomycin inactivation enzyme did not allow the bacteria to grow intracellularly. Third, purified RpsL but not RpsM, another ribosomal protein, induces cell death in macrophages. Fourth, RpsL-induced cell death is independent of caspase 1 or 11, indicating that the potential flagellin [4,5] or LPS [10] contaminants did not contribute to this process. The insensitivity of the RpsL protein preparations to RNase indicates that potential residual RNA [45] is not implicated in the phenotype. Finally, RpsL from other bacteria such as *E*. *coli* was able to trigger macrophage cell death, suggesting that this protein is a universal ligand.

Although RpsL is a highly conserved protein, the distinct cell death phenotype has not yet been observed in infections caused by other intracellular pathogens. Such difference most likely results from the nature of the Dot/Icm type IV transporter of *L*. *pneumophila*, which recognizes hundreds of substrates with diverse primary sequences in the region essential for translocation [12,46]. Although bacteria lysed by macrophages may generate some cytosolic RpsL, we prefer a model in which the relatively promiscuous substrate recognition nature of the Dot/Icm system “accidentally” delivers a sufficient amount of RpsL into the cytosol of macrophages to trigger the response. The importance of the Dot/Icm transporter in this process is further supported by the fact that deletion of its essential component gene *dotA* abolishes the ability of the environmental strain LPE509 to cause cell death [20]. This Dot/Icm-dependent phenotype is akin to that of flagellin, which dictates the susceptibility of mouse lines to *L*. *pneumophila* infection [17,18]. Flagellin from other bacteria is similarly potent in the activation of the NLRC4 inflammasome but does not exhibit such distinct phenotypes in infections [4,5]. Flagellin has not yet been shown to be translocated into host cells by *L*. *pneumophila* with standard reporters. Similarly, we cannot detect Dot/Icm-dependent delivery of RpsL into host cells. Thus, although these proteins can be robustly sensed by their cognate immune recognition system, the amount delivered by the transporter is beyond the sensitivity of current methods for measuring protein translocation.

RpsL is not the first ribosome component shown to be able to trigger immune responses. In plants, EF-Tu, an accessory ribosomal protein is recognized by the receptor kinases EFR, a member of the PRRs, to induce antibacterial immunity [47]. In mice, sensing the 23S rRNA via TRL13 leads to the induction of cytokines such as interleukin-1β [48]. The protozoan profilin-like protein, a non-ribosomal yet integral intracellular component of microorganisms has also been established as the ligand recognized by TLR11 [49].

It is worth noting that methylation of an adenosine in the antibiotic-binding site of 23S rRNA camouflages it from TLR13, and bacteria carrying this modification are resistant to antibiotics such as erythromycin [48]. Similar to this observation, Lysine 88 of RpsL is directly involved in binding to streptomycin, and the mutation of this residue to arginine confers resistance to the antibiotic [21]. Clearly this mutation also renders RpsL less recognizable by macrophages, probably by altering the charging status, conformation or a combination of both. Consistent with this notion, nonpathogenic *E*. *coli* strains harboring *rpsL*_K88R_ survived significantly better than their wild type counterparts in macrophages [50], likely due to less immune activation by RpsL_K88R_ from mutant bacteria. The K88R mutation in RpsL may represent an evolved mechanism that enables the bacteria to gain resistance to both antibiotics and the innate immunity. Alternatively, antibiotics and host innate immune sensors could have converged to recognize the same constrained epitope of the pathogen.

The lysosome is emerging as a signal integration center for various stresses, including those that lead to cell death [38]. Our results indicate that sensing of RpsL leads to the activation of the lysosomal cell death pathway and the induction of LMP, which in turn releases the lysosomal hydrolases, particularly cathepsin B. In agreement with this notion, the cell death cannot be blocked by inhibitors of necrosis or classical apoptosis [20] but can be abrogated by agents targeting vacuolar acidification or cathepsins. LMP can be caused by diverse molecules such as reactive oxygen species (ROS), lipids and certain activated receptors such as TNF-α [38]. It is becoming evident that lysosomes do not indiscriminately release their contents, but rather, certain signals cause selective release of proteins such as cathepsins. For example, selective release of CtsB in response to different signals in different contexts leads to either tumor growth or apoptosis [51–54]. In neutrophils, cytosolic cathepsin D plays a major role in activating caspase-8 to resolve inflammation [55]. One challenge for future studies is to determine the mechanisms that govern the selectivity of cathepsin release in response to specific cues such as RpsL.

The inability of BMDMs from *ctsB* ^-/-^ mice to support intracellular replication of *L*. *pneumophila* strains expressing wild type RpsL is consistent with the apparent multiple effects of CtsB in cell death, including its role in the amplification of events of apoptosis [38] and, in some cell types, the timing of apoptosis [56]. It also agrees well with the fact that the plasma membranes of BMDMs from *ctsB* ^-/-^ can still be damaged after RpsL is introduced. CtsB itself is capable of directly causing cell death directly or by serving as a signal amplifier, which has been documented in TNF-α associated LMP [57]. Alternatively, in addition to cell death, sensing RpsL by BMDMs may activate other yet unidentified pathways that lead to the arrest of bacterial replication. This potential multifaceted mechanism of action differs from that of a terminal cell death enzyme such as caspase-1, whose absence leads to limited growth of *L*. *pneumophila* strains expressing flagellin [58]. It is also possible that the arrest of bacterial replication occurs prior to macrophage death. One focus of the future studies will be the mechanism underlying both the cell death and the intracellular replication phenotypes.

That the cell death occurs only in primary mouse macrophages suggests that RpsL does not directly damage the lysosomal membranes and that its putative receptor clearly is more abundant in or specifically functions in these cells. Whether similar responses occurs in immortalized mouse macrophage lines remains to be determined. Furthermore, it is possible that the pathway activated by RpsL exists only in mouse cells, perhaps because human cells are defective in one or more components of the pathway such as the putative receptor. Such potential differences may explain the fact that *L*. *pneumophila* strains expressing wild type RpsL are able to cause successful infections in humans. It is likely that RpsL engages the putative receptor to activate or to initiate the production of molecules capable of causing LMP. That RpsL-induced macrophage death occurs independent of several inflammatory caspases is consistent with the observation that we did not detect significant induction of inflammatory cytokines in macrophages upon RpsL delivery. The lysosomal cell death process is involved in the activation of the NLRC4 and NLRP3 inflammasomes that are induced by flagellin and cholesterol crystals, respectively [36,59]; whether RpsL-induced cell death is accompanied by any inflammation awaits further investigation. Clearly, identification of the putative receptor for RpsL will greatly facilitate the elucidation of the signaling pathway that leads to LMP. Such results will also aid in the study of the mechanism underlying the differences between mouse and humans in their response to wild type *L*. *pneumophila*.

## Materials and Methods

### Ethics statement

Primary murine macrophages were prepared from indicated mice lines, and the experiments were performed in strict accordance with the regulations of the Public Health Services (PHS) Policy on Humane Care and Use of Laboratory Animals. All animal procedures were performed according to a protocol approved by the Purdue Animal Care and Use Committee (protocol number: 04–081).

### Bacterial strains, plasmids and media

*Legionella pneumophila* strain Philadelphia 1 [24], a generous gift from Dr. Isberg (Tufts Medical School, Boston, MA), its derivatives Lp02 (*dot/icm* ^+^) (Lp02_rpsLK88R_) [24], Lp03(*dotA* ^-^) (_rpsLK88R_) [24], JR32 [25] were used. Strains Paris [27] and Thunder Bay [28] were from Dr. Alex W. Ensminger (University of Toronto). For strains Lp02 and Lp03 and their derivatives that are thymidine auxotrophic [24], we introduced plasmid pJB908 expressing *thyA* [60] to make them thymidine autotrophic for all experiments. The environmental strain LPE509 was described earlier [20]. *L*. *pneumophila* was cultured on charcoal-yeast extract (CYE) plates or in ACES-buffered yeast extract (AYE) broth [61]. *Escherichia coli* strains were grown on L-agar plates or L broth, antibiotics were used at the following concentrations: For *E*. *coli*, Amp, 100 μg/ml; Km, 30 μg/ml; streptomycin 50 μg/ml. For *L*. *pneumophila*, Km was used at 20 μg/ml, Amp was used at 100 μg/ml and streptomycin was used at 100 μg/ml.

Spontaneous streptomycin resistant mutants were isolated by plating cultures of strain LPE509 onto AYE plates containing 100 μg/ml streptomycin. Colonies from independent cultures were purified and used for described experiments. The *rpsL* locus of each mutant was amplified by PCR and the PCR products were sequenced to determine the mutation. The sequence of the 16S rRNA was also determined via similar methods.

Allele exchange of the *rpsL* gene was performed using the standard method [62]. Briefly, the open reading frame of *rpsL* (lpg0324) together with a 300-bp flanking sequence was amplified from genomic DNA of wild type or *rpsL*_K88R_ mutant strain of LPE509. The DNA digested with appropriate restriction enzymes was ligated to the *pir* protein-dependent plasmid pSR47s [63], to produce pZL*rpsL* and pZL*rpsL*_K88R_, respectively. The primers used were: 5′-CTGGAGCTCAAAAGAAAACGTGATGGTAG-3′ and 5′-CTGGTCGACGTACTTCCA CACTTGGGCGA-3′. Plasmids were introduced into appropriate *L*. *pneumophila* strains by electroporation, and transformants were streaked onto CYE plate supplemented with 5% sucrose and the desired strains were screened by the gain or loss of resistance to streptomycin followed by DNA sequencing of the locus.

### Mice, bone marrow-derived macrophage, cell culture and bacterial infection

A/J and C56BL/6 mice were purchased from the Jackson laboratory (Bar Harbor, Maine). Caspase 1/11^-/-^ [64] and the corresponding C56BL/6 control mice were similarly maintained. Mice lacking caspase 1, 7 and 11 were generated by intercrossing Casp1/11^-/-^ mice [65] and Casp7-/-mice [66] (purchased from Jackson Labs, Bar Harbor, ME). The cathepsin B [52] and cathepsin H [67] knock mice (*cstB* ^*-/-*^, *cstH* ^*-/-*^) and the control mice were maintained in the animal facility of New York University School of Medicine. Bone marrow-derived macrophages were prepared from 6–10-week old female mice with L-supernatant as described previously [68]. Macrophages were seeded in 24-well plate the day before infection. Cell density of 4x10^5^/well was used for intracellular bacterial growth and LDH release assay, whereas 2x10^5^/well was used for immunofluorescence staining and other single cell-based assays. *L*. *pneumophila* used for infection was grown in AYE broth to post-exponential phase based on both optical value (OD_600_ = 3.4–4.0) and bacterial motility.

### Intracellular bacterial growth

For intracellular growth curve in mouse macrophages, we infected the cells plated on 24-well at a multiplicity of infection (MOI) of 0.05. Two hours after adding the bacteria, we synchronized the infection by washing the cells with PBS for three times. Infected macrophages were incubated at 37°C with 5% CO_2_. At the desired time point, the cells were lysed with 0.02% saponin, diluted lysate was plated on CYE plates, and CFU were determined from triplicate infections of each strain.

### Antibodies, immunoblotting and immunostaining of infected macrophages

Mouse macrophages seeded on coverslips were infected with *L*. *pneumophila* at an MOI of 1, extracellular bacteria were removed by washing the cells with PBS for three times. Infections were terminated at appropriate time points by fixing with 4% paraformeldehyde. After fixation, samples were first stained for extracellular or total bacteria using anti-*L*. *pneumophila* antibodies [29] at a dilution of 1:10,000 and appropriate secondary antibodies conjugated to distinct fluorescent dyes. Processed coverslips were mounted on glass slides with an anti-fade reagent (Vector laboratories, CA). Samples were examined by visual inspection with an Olympus IX-81 fluorescence microscope.

For western blotting, anti-cleaved PARP was purchased from Abcam (ab2317). Anti-mouse-BID was purchased from R&D System (MAB860). Anti-cleaved caspase-3 was purchased from Cell Signaling Technology (#9664). Anti-tubulin was purchased from Developmental Studies Hybridoma at University of Iowa.

### TUNEL staining

2x10^5^ mouse macrophages were infected with relevant *L*. *pneumophila* at an MOI of 1, and incubated for 12 hours after infection. Samples were fixed and stained with intracellular and extracellular bacteria as described above, and then stained with TUNEL using the *In Situ* Cell Death Detection Kit (Roche Diagnostics, Indianapolis). Fifty microliter TUNEL reaction mixture was added to each coverslip and incubated at 37°C for 1 hr. After 3x washes with PBS, coverslips were mounted with anti-fade reagent.

### Intracellular protein delivery

Recombinant proteins were purified as previously described [69]. Briefly, recombinant proteins bound to Ni-NTA columns were subjected to extensive organic solvent wash (50x bed volume with wash buffer containing 60% isopropanol) to remove the majority of endotoxin contaminants [29]. β-galactosidase was purchased from Clonetech. If necessary, BMDMs were treated with lysosome acidification inhibitor bafilomycin A1 (20 nM), cathepsin B inhibitor CA-074ME (25 μM), cathepsin D inhibitor pepstatin A (25 μM) or DMSO (vehicle) for 1 hour before protein transfection. The purified proteins were then introduced into macrophages using Lipofectamine 2000 as follows: For transfection of 2x10^5^ macrophages, 12.5-μg recombinant protein suspended in 50-μl RPMI-1640 and 2-μl Lipofectamine 2000 suspended in 50-μl RPMI-1640 were equilibrated at RT for 5 min, then mixed at RT for 30 min. The mixture was then directly applied to the macrophage cultures.

### Acridine orange staining

BMDMs plated on glass coverslips at a density of 2x10^5^/well were either transfected with appropriate proteins or infected with different *L*. *pneumophila* strains at an MOI of 1. For infection, the samples were washed 3 times with warm PBS to synchronize the infection 2 hrs after adding the bacteria. The transfected/infected samples were incubated for 8 hrs. After centrifugation at 200*g* for 5 min, the culture medium was removed, and 0.5 ml of the dye solution containing acridine orange (5 μg/ml) was added into each well. The plate was incubated at 37°C for 15 min before imaging using a fluorescence microscope (Olympus IX-81).

### LDH release assay

LDH release during infection was determined using CytoTox 96 Assay (Promega, Madison, WI). 4x10^5^ BMDMs were either transfected with appropriate proteins or infected with appropriate *L*. *pneumophila* strains at an MOI of 1. Four or six hours post transfection/infection, cell culture was centrifuged at 200*g* for 5 min, and 50-μl supernatant of each well was transferred to a new 96-well enzymatic assay plate. Fifty microliter reconstituted Substrate Mix was then added to each well of enzymatic assay plate, and incubated in dark at room temperature for 30 min. The enzymatic reaction was stopped by adding 50-μl stop solution to each well. The absorbance at 490 nm was measured using a Biotek microplate reader. Total LDH release was determined by complete lysis of the cells using a lysis solution provided by the manufacturer, and spontaneous LDH release was determined by using the supernatant of cells without infection/transfection. The percentage of LDH release was calculated with the follow formula: LDH release (%) = (Experimental LDH release-Spontaneous LDH release)/(Total LDH release-Spontaneous LDH release)x100.

### Cytosolic cathepsin activity

4×10^5^ BMDMs from A/J mice were plated in 24-well plates. RpsL proteins were delivered into the cells as described above. 6 hours post protein transfection, cytosolic cathepsin B activity was determined as previously described [70]. Briefly, macrophages were washed with phenol red and HEPES free DMEM (Invitrogen, OR). 100μg/ml saponin was added to each well and samples are incubated for 10 min on ice. Cell lystae was cleared at 500xg for 10 min at 4°C in flat-bottom 96-well plates. 10 μl of the cell lysate was then added to 90 μl of the cathepsin B substrate buffer (100 μM zRR-AMC, 50 mM sodium acetate [pH 6.0], 4 mM EDTA, 10 mM dithiothreitol, 1 mM Pefablock). The generation of free AMC was determined by recording fluorescence (excitation, 355 nm; emission, 460 nm) at 30° in a TECAN multiplate reader. Cathepsin B activity was determined by measuring the increase in AMC fluorescence. Aspartic cathepsin activity was determined by measuring the aspartic cathepsin activity in total (0.02% Triton) or cytosolic fraction using Mca-KPLGL-Dpa-AR-NH2 Fluorogenic Peptide (R&D, cat# ES010), in a buffer containing 0.1 M NaOAc and 0.2 M NaCl, pH 3.5.

## Supporting Information

S1 Table The minimal inhibition concentration (MIC) of streptomycin for *L*. *pneumophila* strains. (DOCX)

S1 Fig Intracellular replication of *L*. *pneumophila* streptomycin resistance mutants in mouse bone marrow-derived macrophages. Eight spontaneous streptomycin resistance mutants isolated by plating bacterial cultures onto bacteriological medium grown to post exponential phase were used to infect BMDMs from A/J mice at an MOI of 0.05. After synchronization of the infections 2 hrs post infection, total bacterial counts were determined at the indicated time points. Infections were performed in triplicate and results shown are one of two experiments done independently.
(TIFF)

S2 Fig Resistance to streptomycin did not allow *L*. *pneumophila* strains harboring wild type *rpsL* to replicate in A/J mouse macrophages. A plasmid expressing a streptomycin adenyltransferase that confers resistant to the antibiotic was introduced into *L*. *pneumophila* strain LPE509 and the Lp02 derivative harboring wild type RpsL(Lp02_rpsLWT_). The resulting bacterial strains were used to infect BMDMs from A/J mice together with relevant controls strains at an MOI of 0.05. After synchronization at 2 hrs psi, total bacterial counts (colony-forming-unit) were determined at indicated time points by plating appropriately diluted saponin solubilized infected cells onto bacteriological medium. Infections were performed in triplicate and data shown were from one representative of three independent experiments with similar results.
(TIFF)

S3 Fig Primary macrophages from A/J mice do not support replication of wild type clinic strains. Clinic *L*. *pneumophila* strain Paris, Thunder Bay and relevant control strains were used to infect primary macrophages from A/J mice at an MOI of 0.05. Total bacterial counts were determined following standard procedures. Infections with each strain were performed in triplicate and similar results were obtained in two independent experiments.
(TIFF)

S4 Fig Laboratory strain Lp02 expressing wild type RpsL replicates robustly in two human cell lines. Strain Lp02_rpsLWT_ and Lp02_rpsLK88R_ were used to infect Hela cells (**A**) or the human macrophage cell line U937 (**B**) at an MOI of 0.05. After 2 hrs incubation, infections were synchronized by washing 3 times with warm PBS. Total bacterial counts were determined at indicated time points by spreading appropriately diluted lysates of infected samples onto bacteriological medium. Similar results were obtained in two independent experiments.
(TIFF)

S5 Fig *L*. *pneumophila* strains expressing wild type RpsL is able to form phagosomes with multiple bacteria. BMDMs from A/J mice were infected with the indicated strains of L. pneumophila at an MOI of 1 for 14 hrs and the infected samples were fixed for immunostaining. Extracellular and intracellular bacteria were sequentially labeled Legionella-specific antibodies and secondary antibodies conjugated with distinct fluorophores. The size distribution of the phagosomes was scored by counting the number of bacteria in the vacuoles. Phagosome categories: 1–3 bacteria, small vacuoles; 4–9 bacteria, medium vacuole; more than 10 bacteria, large vacuoles. At least 150 phagosomes were scored from each infection done in triplicate. Similar results were obtained in two independent experiments.
(TIFF)

S6 Fig Viable bacteria of strains expressing wild type RpsL or the K43N mutant decrease in the first infection cycle in macrophages. Indicated *L*. *pneumophila* strains grown to postexponential phase were used to infect BMDMs from A/J mice at an MOI of 0.05. After synchronization at 2 hrs after adding the bacteria to the cell culture, total bacterial counts were determined every 3 hours by spreading saponin lysates of infected samples onto bacteriological medium. Note that only the strain expressing RpsL_K88R_ displayed significant increase in total viable bacterial counts. On the other hand, the number of colony-forming unit for both strain Lp02_rpsLWT_ and Lp02_rpsLK43N_ decreased in the experimental duration.
(TIFF)

S7 Fig Caspase-1, 7 and 11 are dispensable for growth restriction of *L*. *pneumophila* bearing wild type *rpsL*. Bone marrow-derived macrophages from C57L/B6 (**A**) or caspase-1/7/11^-/-^ (**B**) mice were challenged with indicated bacterial strains grown to post-exponential phase at an MOI of 0.05. After synchronization 2 hrs postinfection, total bacterial counts (colony-forming-unit) were determined at indicated time points. Infections were performed in triplicate and data shown were from one representative of three experiments with similar results.
(TIFF)

S8 Fig RpsL is not detectably translocated into host cells by *L*. *pneumophila*. **A**. RpsL and RpsL_K88R_ were fused to β-lactamase on a plasmid, respectively and the resulting constructs were transformed into *L*. *pneumophila* strains. The bacterial strains were used to determine Dot/Icm-dependent protein translocation with the CCF4-AM substrate with an established protocol. Strains expressing RalF or FabI were used as positive and negative controls, respectively. Note the robust translocation by the RalF construct indicated by the appearance of blue cells after bacterial infection. **B**. Expression of the relevant fusions in *L*. *pneumophila*. The bacterial cells used for infection in (**A**) were probed for the expression of the fusion with a β-lactamase specific antibody. The isocitrate dehydrogenase (ICDH) was probed as a loading control.
(TIFF)

S9 Fig RpsL does not induce cell death in Hela cells. Hela cells seeded on coverslips were transfected with His_6_-RpsL, His_6_-RpsL_K88R_ (**A-B**) or their mixture with β-galactosidase (**C**). Samples treated with staurosporine were established as positive controls. The samples were processed for TUNEL staining or for β-galactosidase staining with 5-bromo-4-chloro-3-indolyl-β-D-galactopyranoside (X-gal) (**B-C**) to evaluate the effectiveness of transfection. Assays were performed in triplicate and similar results were obtained in two independent experiments. Note the X-gal staining of transfected β-galactosidase (blue) in C. Bar, 20 μm.
(TIFF)

S10 Fig RpsL induces the release of aspartic cathepsins into the cytosol in BMDMs from *ctsB* ^-/-^ mice. BMDMs from the indicated mouse lines were transfected with RpsL or RpsL_K88R_ for 6 hrs; samples receiving only the transfection reagent were also included as controls. The activity was determined by measuring the aspartic cathepsin activity in total (0.02% Triton) or cytosolic fraction using the Mca-KPLGL-Dpa-AR-NH2 Fluorogenic Peptide (R&D Systems, cat# ES010). Similar results were obtained in two independent experiments.
(TIFF)

S11 Fig BMDMS from ctsB^-/-^ mice do not support intracellular replication of *L*. *pneumophila* strains expressing wild type RpsL. Bone marrow-derived macrophages from C57L/B6 (**A**), *ctsB* ^-/-^ (**B**) or *ctsH* ^-/-^ (**C**) mice were challenged with indicated bacterial strains grown to post-exponential phase at an MOI of 0.05. After synchronization 2 hrs postinfection, total bacterial counts (colony-forming-unit) were determined at indicated time points. Infections were performed in triplicate and data shown were from one representative of three experiments with similar results.
(TIFF)
