# Supplementary material for: Sensing Cytosolic RpsL by Macrophages Induces Lysosomal Cell Death and Termination of Bacterial Infection
Source: PLoS Pathog. 2015 Mar 4;11(3):e1004704. doi: 10.1371/journal.ppat.1004704 (PMC4349785; doi:10.1371/journal.ppat.1004704)
Supplement: S1 Table — (DOCX) [file ppat.1004704.s001.docx]

Table S1 The minimal inhibition concentration (MIC) of streptomycin for *L. pneumophila* strains

| **Strains** | **Streptomycin MIC_50_ (mg/L)** |
| --- | --- |
|  |  |
| LPE509*rpsL*_WT_ | 0.16 |
| LPE509*rpsL*_K88R_ | >100 |
| LPE509*rpsL*_K43N_ | >100 |
| LPE509*rpsL*_WT_+pRG970 | 30 |
| Lp02*rpsL*_WT_ | 0.16 |
| Lp02 *rpsL*_K88R_ | >100 |
| Lp02 *rpsL*_WT_+pRG970 | 30 |
|  |  |
