# Supplementary figures and images for: Sensing Cytosolic RpsL by Macrophages Induces Lysosomal Cell Death and Termination of Bacterial Infection
Source: PLoS Pathog. 2015 Mar 4;11(3):e1004704. doi: 10.1371/journal.ppat.1004704 (PMC4349785; doi:10.1371/journal.ppat.1004704)

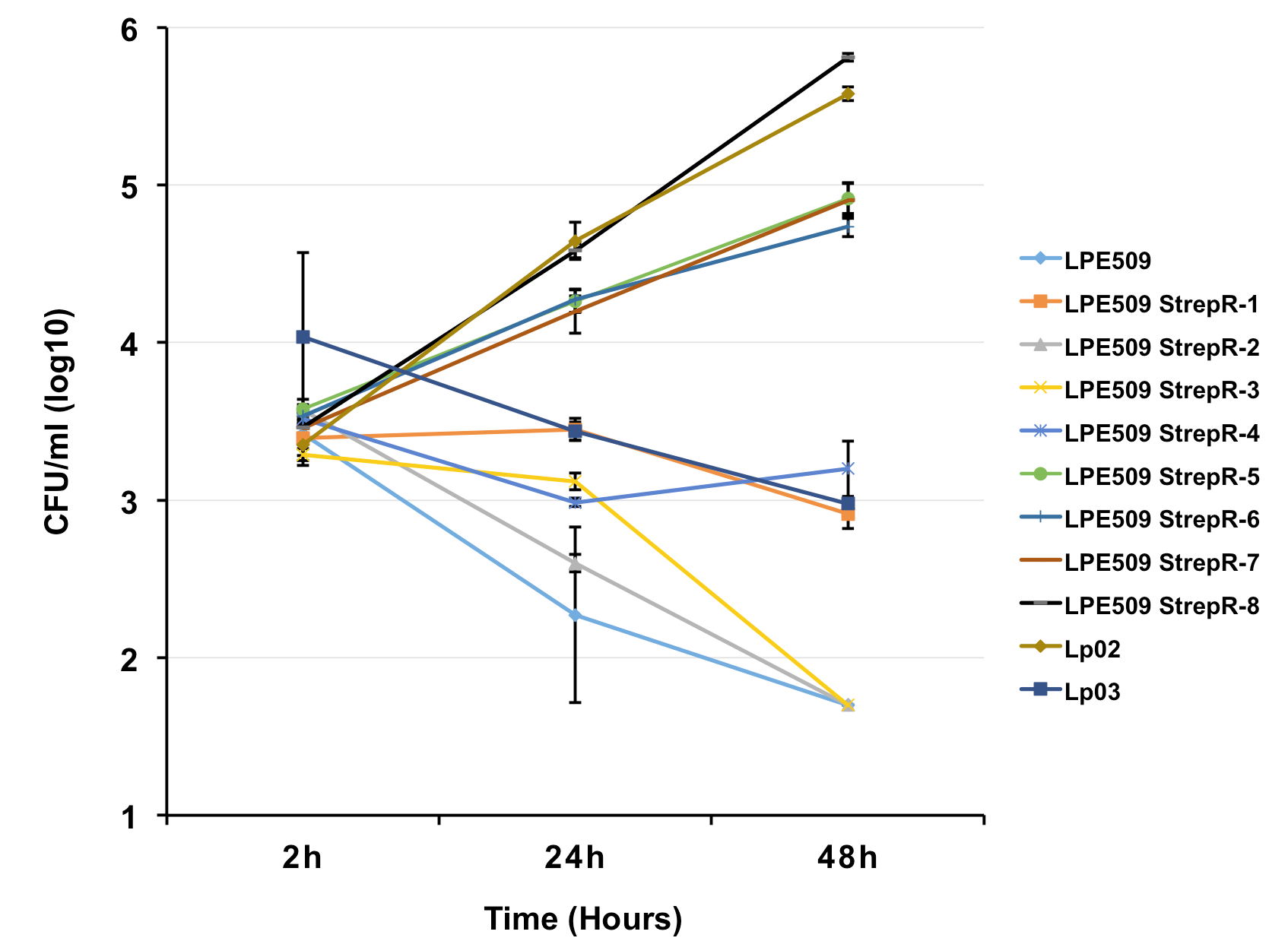

Supplement: S1 Fig — Eight spontaneous streptomycin resistance mutants isolated by plating bacterial cultures onto bacteriological medium grown to post exponential phase were used to infect BMDMs from A/J mice at an MOI of 0.05. After synchronization of the infections 2 hrs post infection, total bacterial counts were determined at the indicated time points. Infections were performed in triplicate and results shown are one of two experiments done independently. (TIFF) [file ppat.1004704.s002.tiff]

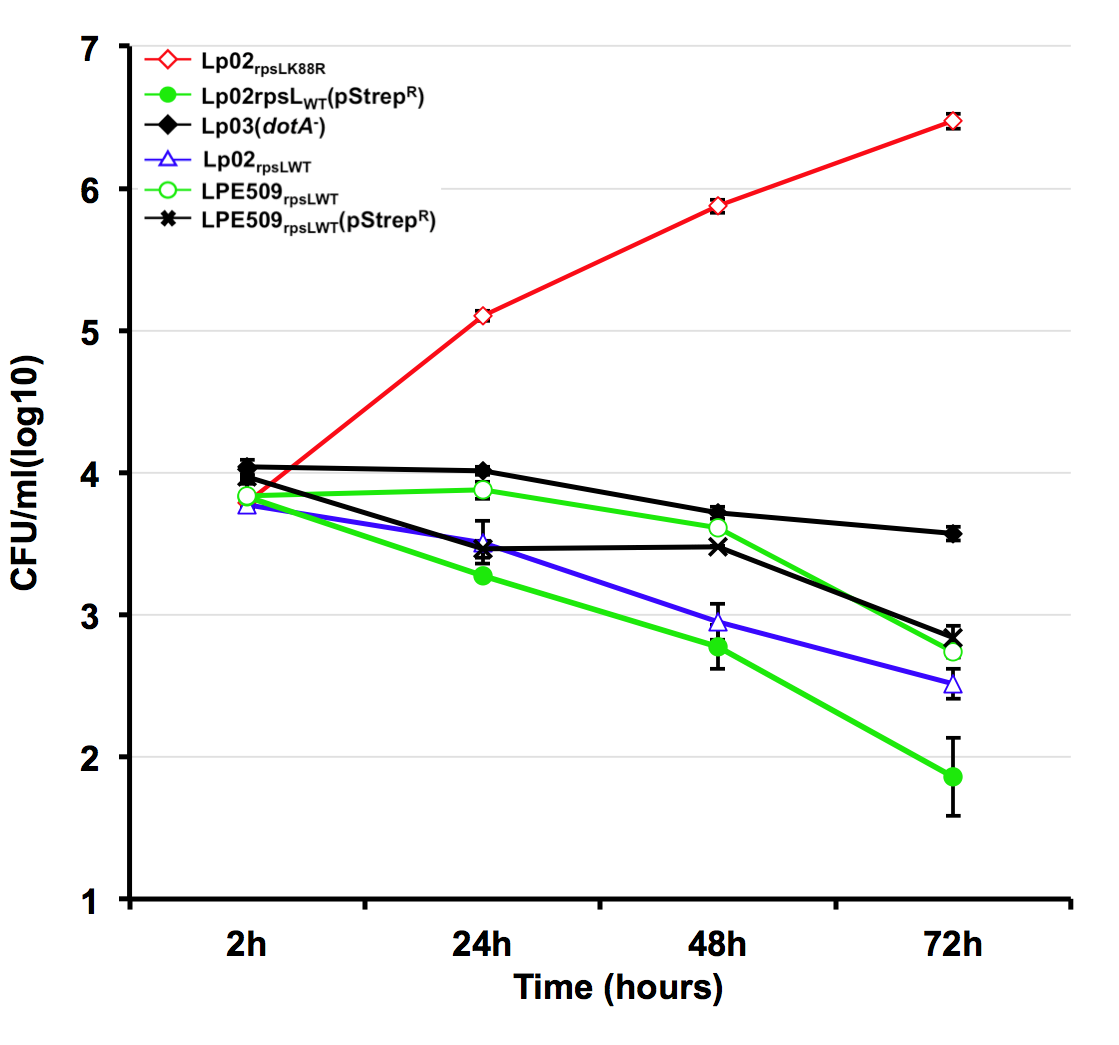

Supplement: S2 Fig — A plasmid expressing a streptomycin adenyltransferase that confers resistant to the antibiotic was introduced into L. pneumophila strain LPE509 and the Lp02 derivative harboring wild type RpsL(Lp02rpsLWT). The resulting bacterial strains were used to infect BMDMs from A/J mice together with relevant controls strains at an MOI of 0.05. After synchronization at 2 hrs psi, total bacterial counts (colony-forming-unit) were determined at indicated time points by plating appropriately diluted saponin solubilized infected cells onto bacteriological medium. Infections were performed in triplicate and data shown were from one representative of three independent experiments with similar results. (TIFF) [file ppat.1004704.s003.tiff]

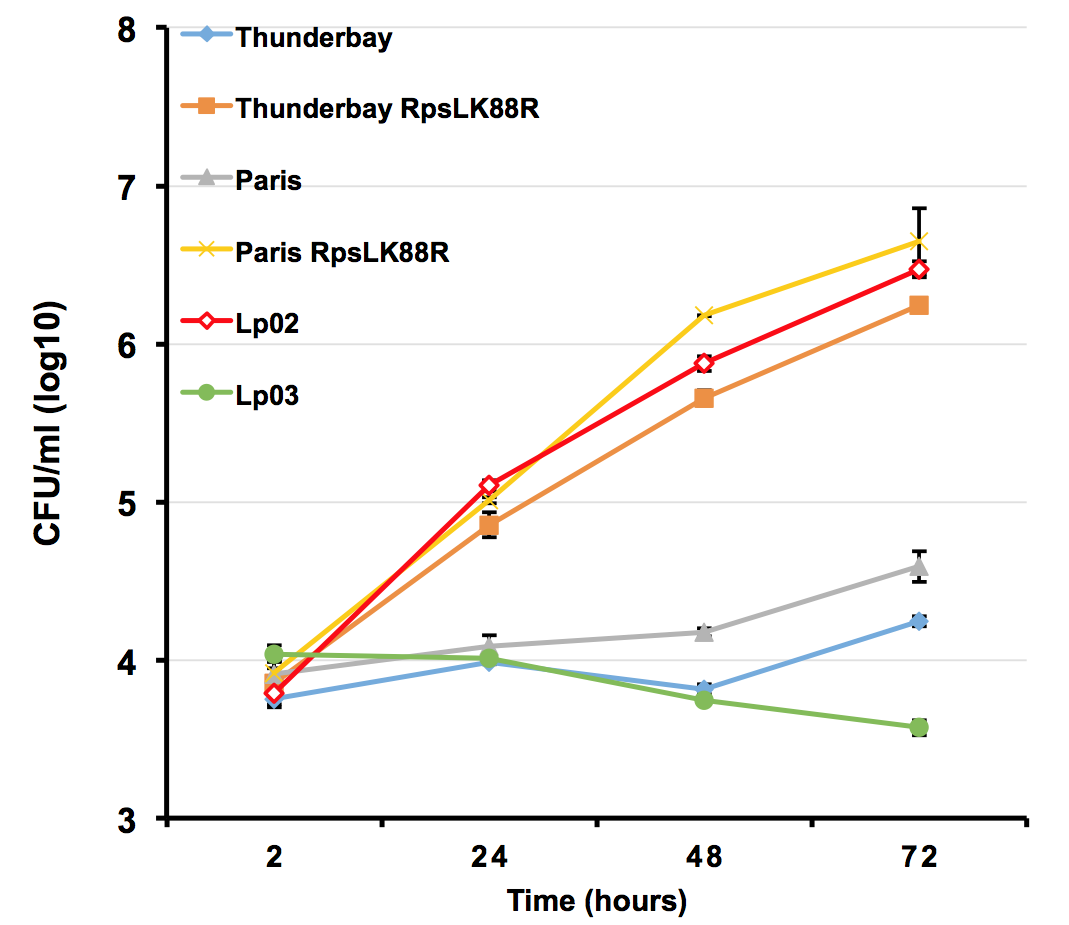

Supplement: S3 Fig — Clinic L. pneumophila strain Paris, Thunder Bay and relevant control strains were used to infect primary macrophages from A/J mice at an MOI of 0.05. Total bacterial counts were determined following standard procedures. Infections with each strain were performed in triplicate and similar results were obtained in two independent experiments. (TIFF) [file ppat.1004704.s004.tiff]

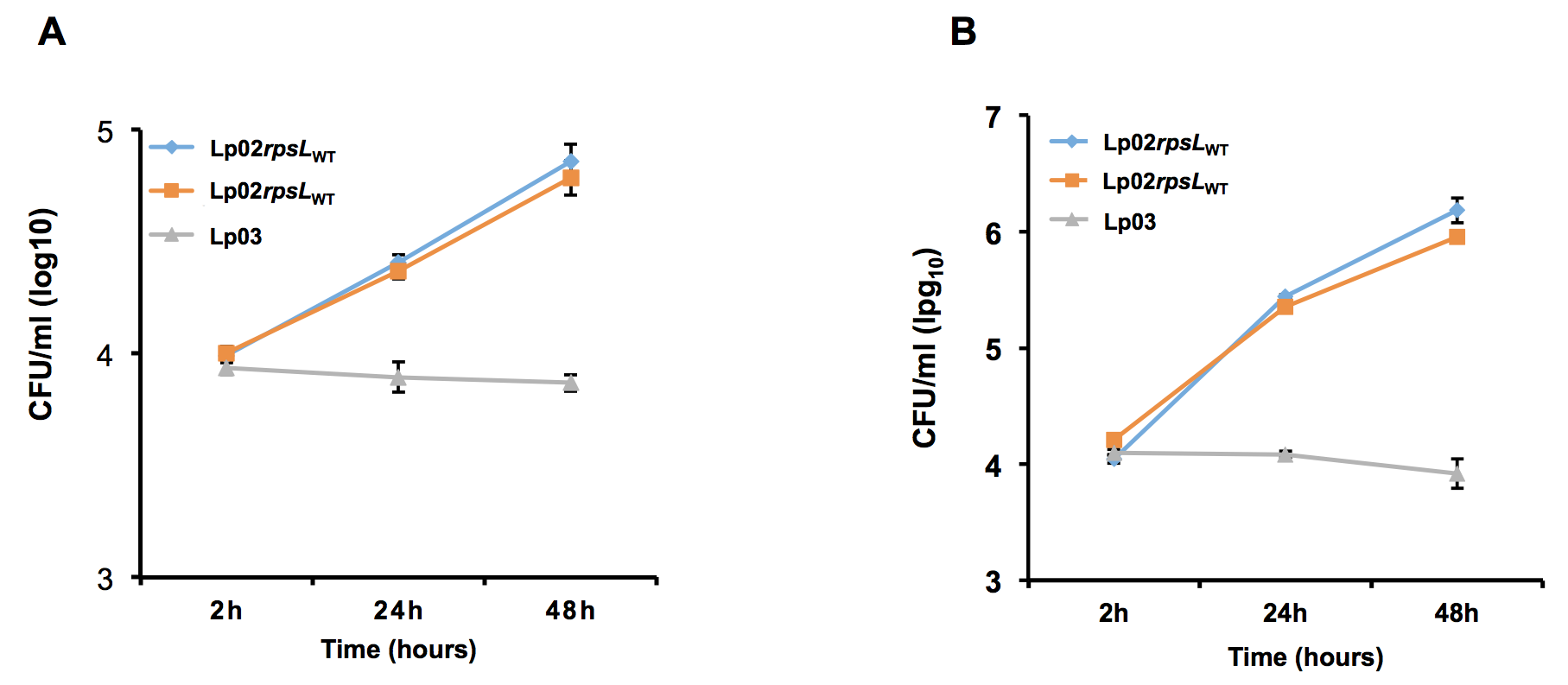

Supplement: S4 Fig — Strain Lp02rpsLWT and Lp02rpsLK88R were used to infect Hela cells (A) or the human macrophage cell line U937 (B) at an MOI of 0.05. After 2 hrs incubation, infections were synchronized by washing 3 times with warm PBS. Total bacterial counts were determined at indicated time points by spreading appropriately diluted lysates of infected samples onto bacteriological medium. Similar results were obtained in two independent experiments. (TIFF) [file ppat.1004704.s005.tiff]

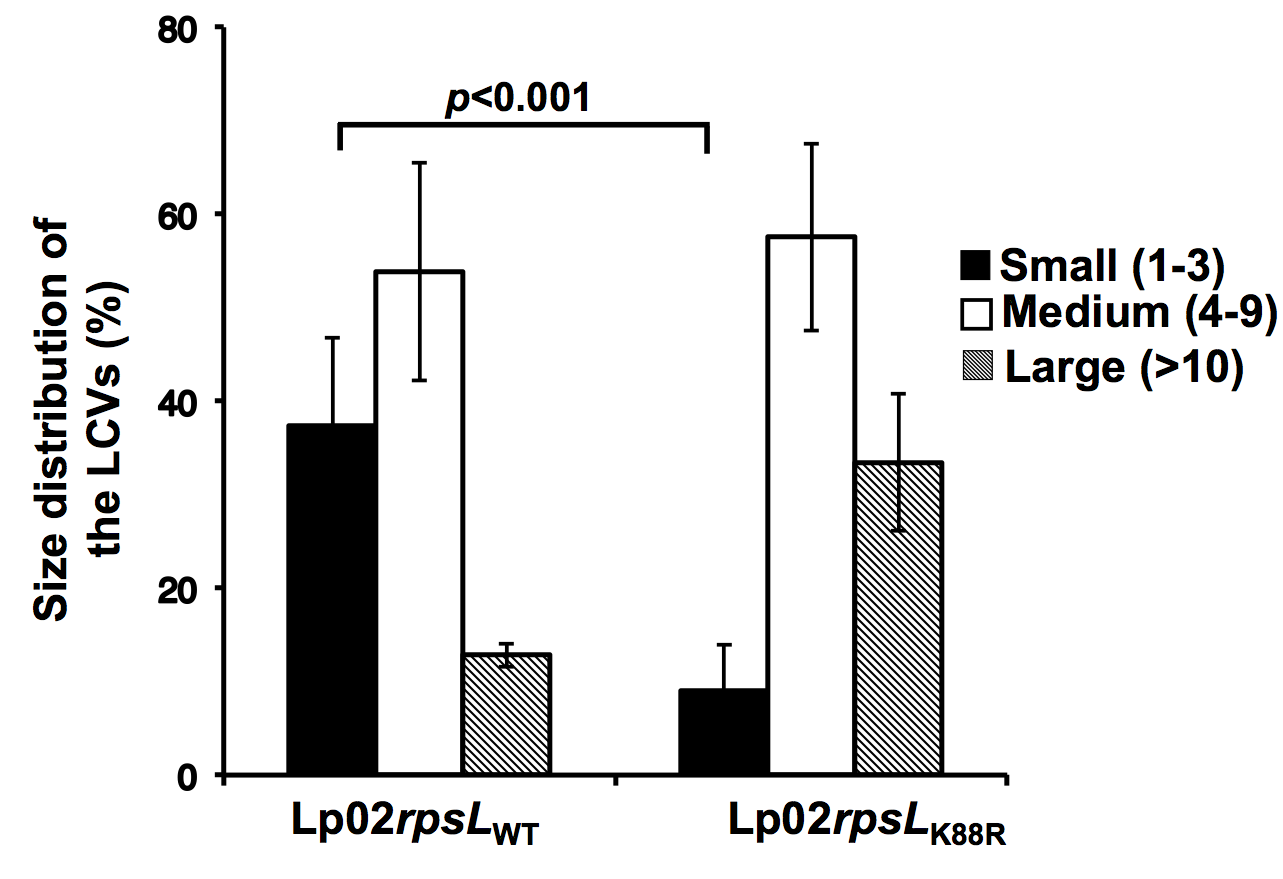

Supplement: S5 Fig — BMDMs from A/J mice were infected with the indicated strains of L. pneumophila at an MOI of 1 for 14 hrs and the infected samples were fixed for immunostaining. Extracellular and intracellular bacteria were sequentially labeled Legionella-specific antibodies and secondary antibodies conjugated with distinct fluorophores. The size distribution of the phagosomes was scored by counting the number of bacteria in the vacuoles. Phagosome categories: 1–3 bacteria, small vacuoles; 4–9 bacteria, medium vacuole; more than 10 bacteria, large vacuoles. At least 150 phagosomes were scored from each infection done in triplicate. Similar results were obtained in two independent experiments. (TIFF) [file ppat.1004704.s006.tiff]

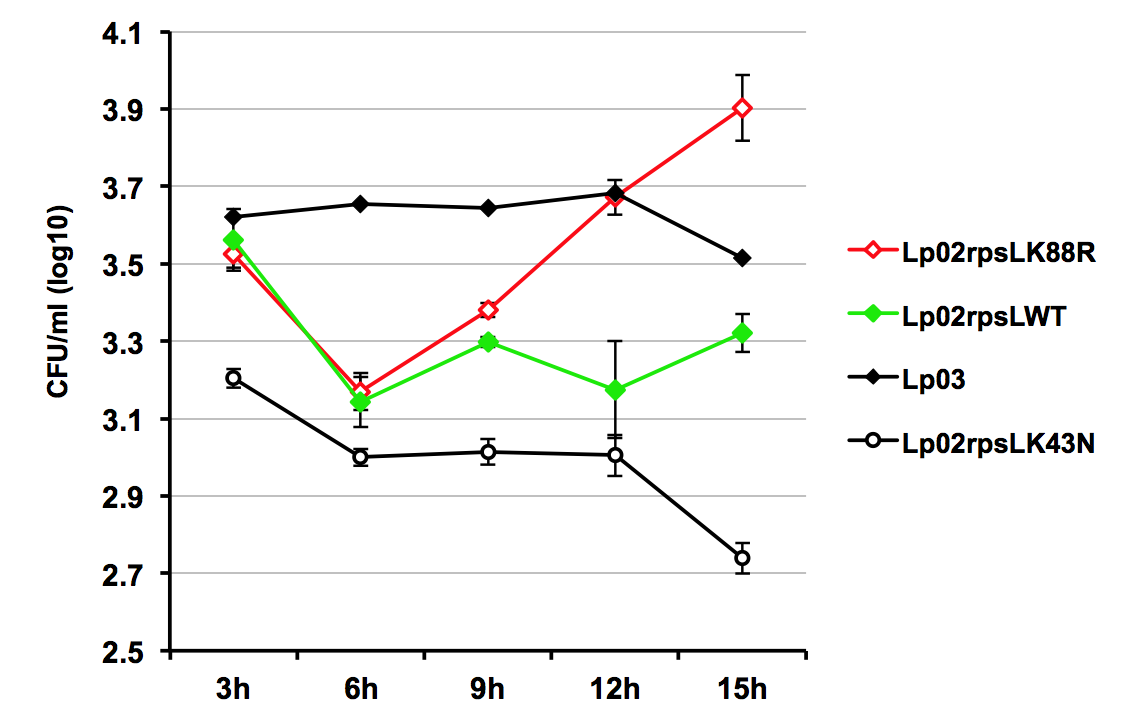

Supplement: S6 Fig — Indicated L. pneumophila strains grown to postexponential phase were used to infect BMDMs from A/J mice at an MOI of 0.05. After synchronization at 2 hrs after adding the bacteria to the cell culture, total bacterial counts were determined every 3 hours by spreading saponin lysates of infected samples onto bacteriological medium. Note that only the strain expressing RpsLK88R displayed significant increase in total viable bacterial counts. On the other hand, the number of colony-forming unit for both strain Lp02rpsLWT and Lp02rpsLK43N decreased in the experimental duration. (TIFF) [file ppat.1004704.s007.tiff]

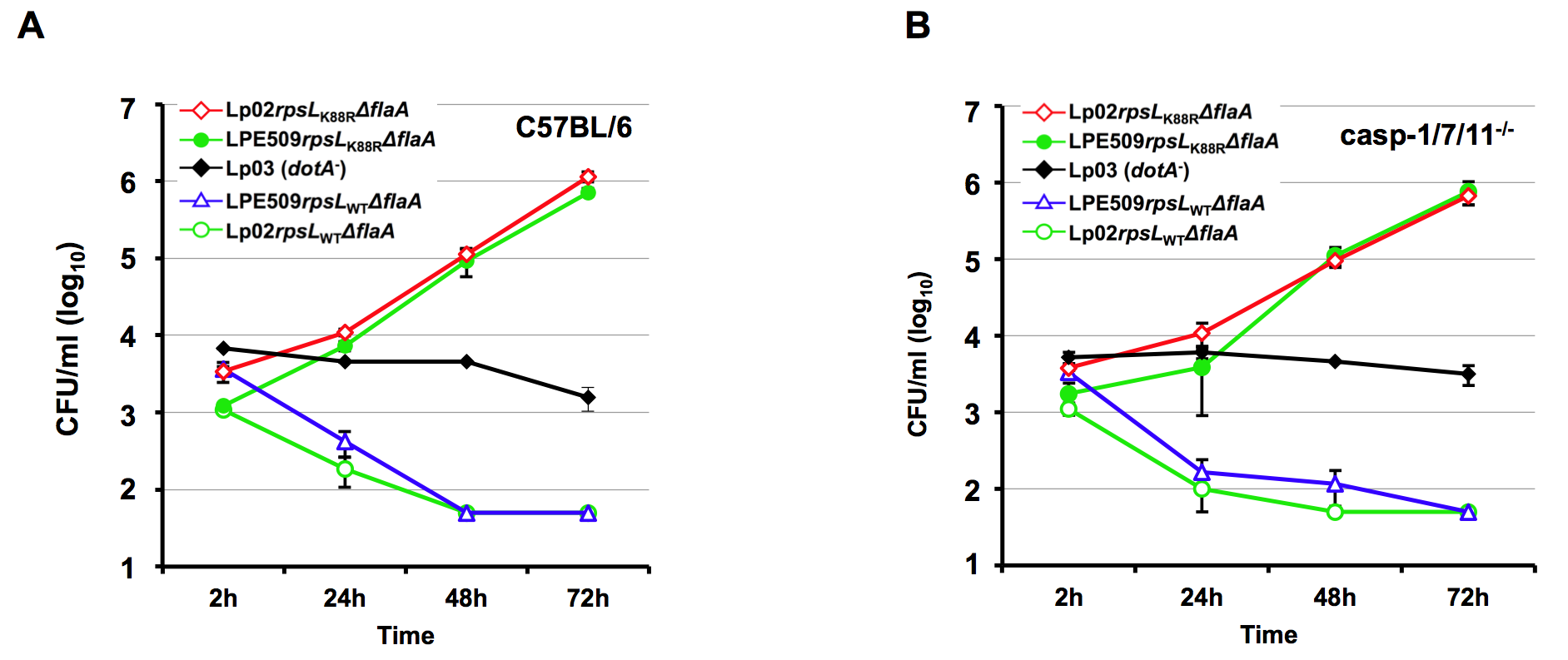

Supplement: S7 Fig — Bone marrow-derived macrophages from C57L/B6 (A) or caspase-1/7/11-/- (B) mice were challenged with indicated bacterial strains grown to post-exponential phase at an MOI of 0.05. After synchronization 2 hrs postinfection, total bacterial counts (colony-forming-unit) were determined at indicated time points. Infections were performed in triplicate and data shown were from one representative of three experiments with similar results. (TIFF) [file ppat.1004704.s008.tiff]

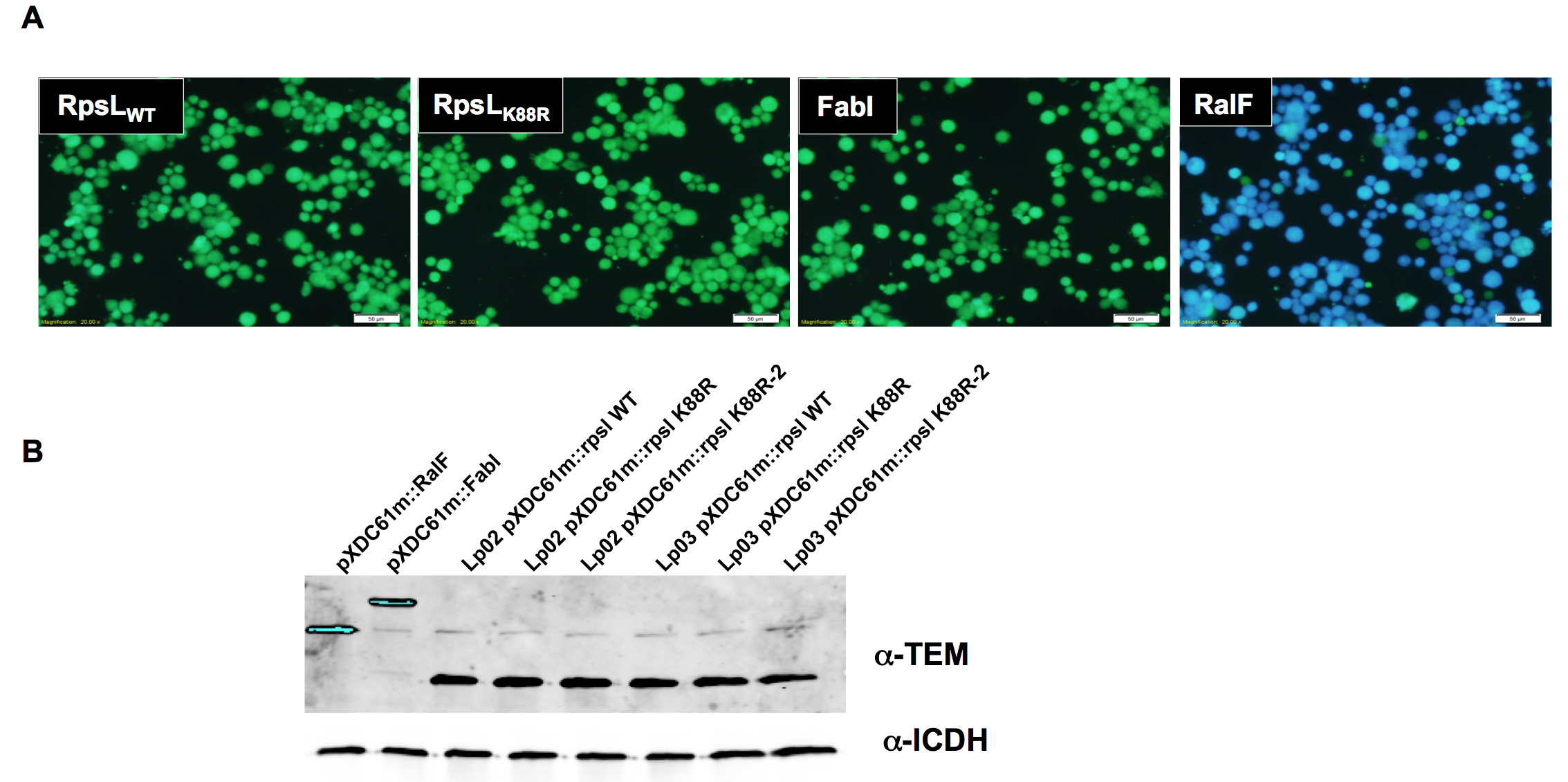

Supplement: S8 Fig — A. RpsL and RpsLK88R were fused to β-lactamase on a plasmid, respectively and the resulting constructs were transformed into L. pneumophila strains. The bacterial strains were used to determine Dot/Icm-dependent protein translocation with the CCF4-AM substrate with an established protocol. Strains expressing RalF or FabI were used as positive and negative controls, respectively. Note the robust translocation by the RalF construct indicated by the appearance of blue cells after bacterial infection. B. Expression of the relevant fusions in L. pneumophila. The bacterial cells used for infection in (A) were probed for the expression of the fusion with a β-lactamase specific antibody. The isocitrate dehydrogenase (ICDH) was probed as a loading control. (TIFF) [file ppat.1004704.s009.tiff]

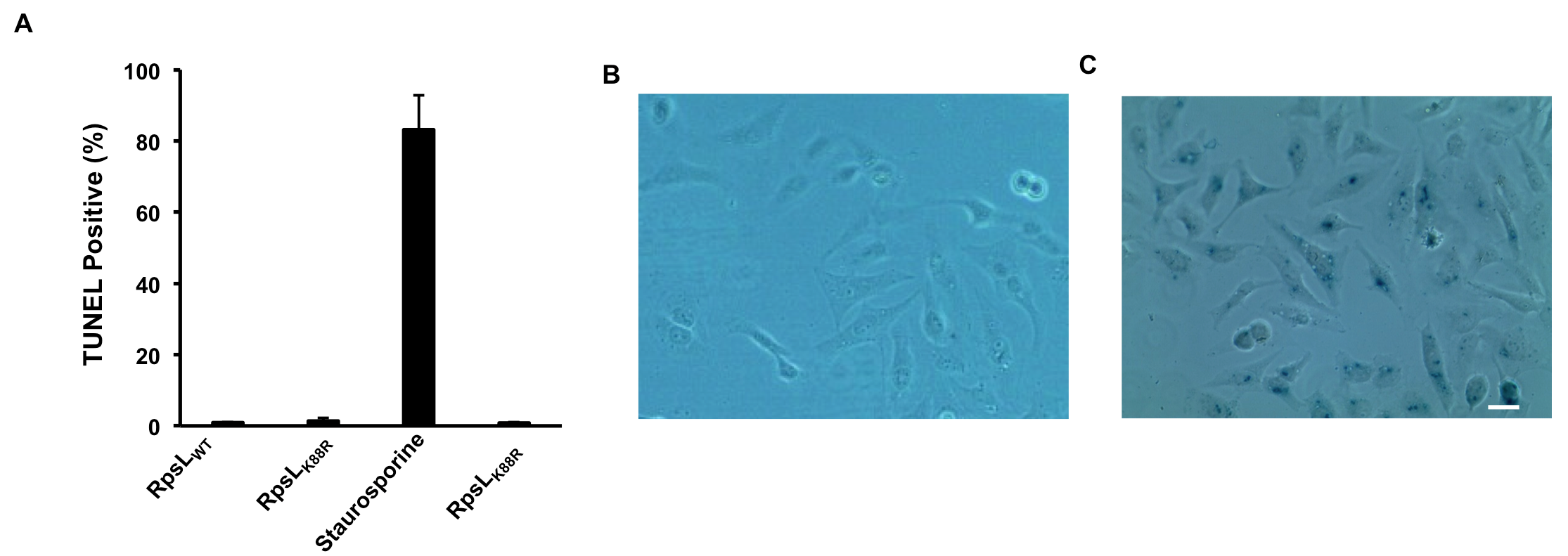

Supplement: S9 Fig — Hela cells seeded on coverslips were transfected with His6-RpsL, His6-RpsLK88R (A-B) or their mixture with β-galactosidase (C). Samples treated with staurosporine were established as positive controls. The samples were processed for TUNEL staining or for β-galactosidase staining with 5-bromo-4-chloro-3-indolyl-β-D-galactopyranoside (X-gal) (B-C) to evaluate the effectiveness of transfection. Assays were performed in triplicate and similar results were obtained in two independent experiments. Note the X-gal staining of transfected β-galactosidase (blue) in C. Bar, 20 μm. (TIFF) [file ppat.1004704.s010.tiff]

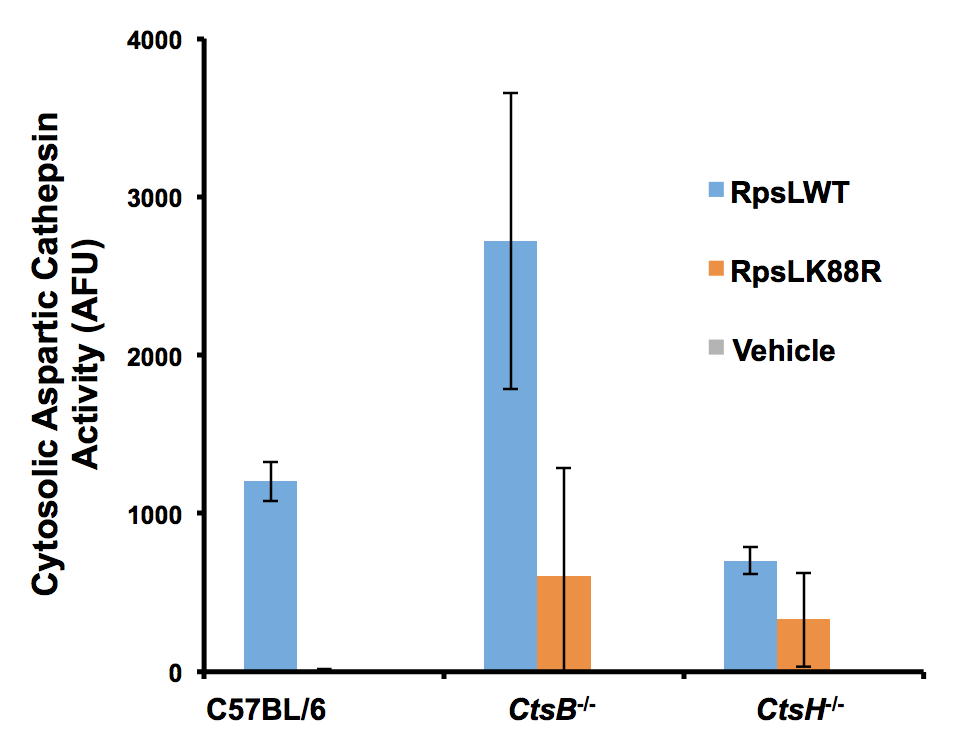

Supplement: S10 Fig — BMDMs from the indicated mouse lines were transfected with RpsL or RpsLK88R for 6 hrs; samples receiving only the transfection reagent were also included as controls. The activity was determined by measuring the aspartic cathepsin activity in total (0.02% Triton) or cytosolic fraction using the Mca-KPLGL-Dpa-AR-NH2 Fluorogenic Peptide (R&D Systems, cat# ES010). Similar results were obtained in two independent experiments. (TIFF) [file ppat.1004704.s011.tiff]

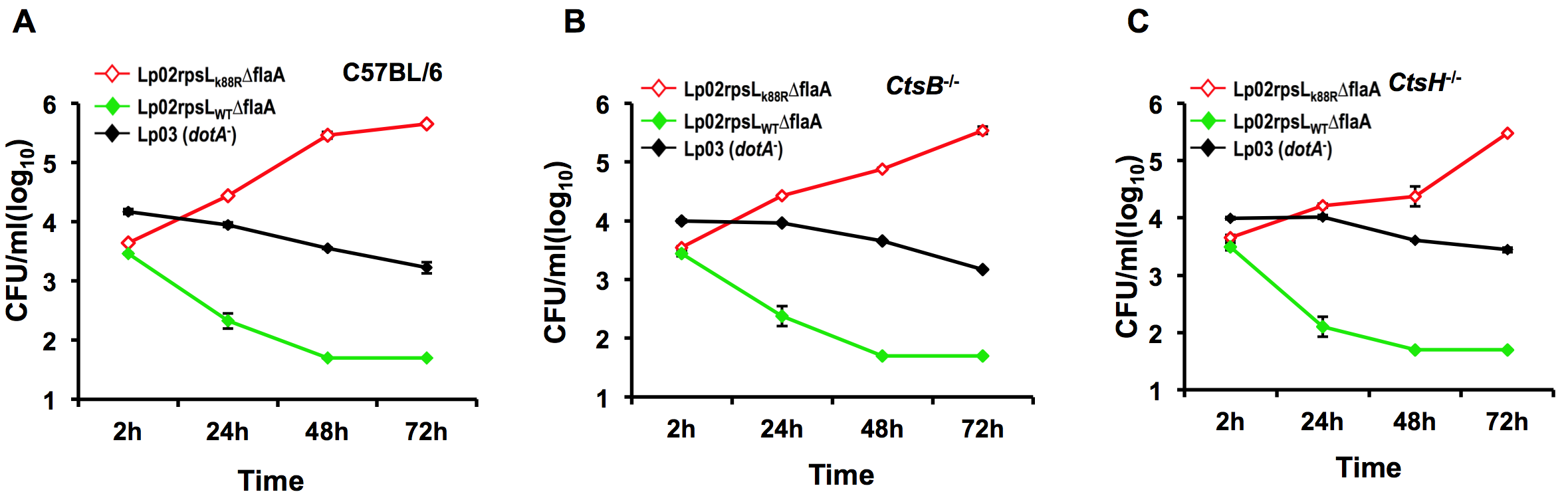

Supplement: S11 Fig — Bone marrow-derived macrophages from C57L/B6 (A), ctsB -/- (B) or ctsH -/- (C) mice were challenged with indicated bacterial strains grown to post-exponential phase at an MOI of 0.05. After synchronization 2 hrs postinfection, total bacterial counts (colony-forming-unit) were determined at indicated time points. Infections were performed in triplicate and data shown were from one representative of three experiments with similar results. (TIFF) [file ppat.1004704.s012.tiff]
